# An individualised Lifestyle Intervention with Physical Activity and Diet in individuals with overweight and obesity (LI-PAD)—study protocol of a 6-month randomised controlled study {1a}

**DOI:** 10.1186/s13063-026-09606-6

**Published:** 2026-03-16

**Authors:** Carina U. Persson, Daniel Arvidsson, Jonatan Fridolfsson, Göran Bergström, Christina E. Lundberg, Mats Börjesson

**Affiliations:** 1 Centre for Lifestyle Intervention, Department of Medicine Geriatrics and Emergency Medicie/Östra (MGAÖ), Sahlgrenska University Hospital, Region Västra Götaland, Gothenburg, Sweden; 2 Department of Molecular and Clinical Medicine, Institute of Medicine, Sahlgrenska Academy, University of Gothenburg, Gothenburg, Sweden; 3 Department of Occupational Therapy and Physiotherapy, Sahlgrenska University Hospital, Region Västra Götaland, Gothenburg, Sweden; 4 Department of Clinical Neuroscience, Institute of Neuroscience and Physiology, Rehabilitation Medicine, Sahlgrenska Academy, University of Gothenburg, Gothenburg, Sweden; 5 Department of Food and Nutrition, and Sport Science, Faculty of Education, University of Gothenburg, Gothenburg, Sweden; 6 Department of Clinical Physiology, Sahlgrenska University Hospital, Region Västra Götaland, Gothenburg, Sweden

**Keywords:** Obesity, Overweight, Weight loss, Health behaviour, Healthy lifestyle, Health promotion, Primary prevention, Behaviour therapy, Diet, Exercise

## Abstract

**Background:**

Overweight and obesity increase the risk of cardiometabolic disease, and sustainable lifestyle change remains difficult to achieve. The primary objective of this randomised controlled trial will be to identify whether a 6-month individualised intervention in physical activity and diet, offering flexible support strategies, is effective and feasible and leads to larger improvements in weight loss, other cardiometabolic disease risk factors, and health-related quality of life compared with standard written lifestyle advice.

**Methods:**

Eligible participants will be 45–65 years old men and women residing in the Gothenburg area, with a body mass index of ≥28 and <35. We intend to recruit 120 females and males, who are randomly allocated in a 1:1 ratio to either the intervention or control group. The intervention group will undergo a 6-month intervention of individually tailored support and guidance on lifestyle behaviours encompassing physical activity and dietary modifications. The control group will receive standard lifestyle advice based on general physical activity and dietary recommendations, respectively. The primary outcome measure will be weight loss from baseline to the 6-month follow-up. The study will be conducted at the Centre for Lifestyle Intervention, Sahlgrenska University Hospital, Gothenburg, Sweden. Data analysis will employ multilevel mixed modelling for repeated measures, alongside linear and logistic analyses, adhering to an intention-to-treat approach.

**Discussion:**

By addressing critical health metrics, such as weight, blood pressure, and physical activity, using individualised advice based on objective data, this intervention will move beyond a one-size-fits-all strategy when tackling complex behaviour change challenges in overweight and obesity management and reducing cardiometabolic disease risk. Introducing health promoters in a clinical setting will be a novel approach.

**Trial registration:**

The study is prospectively registered on researchweb.org on 2 January 2024 (project number 281907) and subsequently at ClinicalTrials.gov, NCT06379802 on 9 February 2024 (approval number/ID: 2023-00546-01).

**Supplementary Information:**

The online version contains supplementary material available at 10.1186/s13063-026-09606-6.

## Introduction

### Background and rationale {9a}

The global prevalence of overweight and obesity has risen dramatically in recent decades, with worldwide rates nearly tripling since 1975 [1, 2]. Recent data from the USA indicate an adult obesity prevalence of 40.3% [3], whilst in Europe, more than 60% of adults have overweight or obesity [4]. This obesity epidemic is alarming due to its strong association with numerous chronic diseases [5–7]. In Sweden, obesity has emerged as a pressing health issue, with nearly half of middle-aged adults exhibiting multiple unhealthy lifestyle behaviours related to obesity [8]. Physical inactivity and poor dietary habits are particularly prevalent, affecting approximately 38% of this population [8, 9]. These behaviours are largely shaped by an obesogenic environment through, for example, reduced active transportation opportunities [10] and increased availability of ultra-processed and energy-dense foods [11].

Currently, behaviour change interventions with physical activity and diet are core components for obesity treatment in clinical settings [12, 13], complemented by pharmacological treatments [14] and surgical procedures (e.g. gastric bypass) [15] in individuals with more severe conditions [16]. However, pharmacotherapy and bariatric procedures are often costly and associated with side effects [17, 18]. Achieving sustained behavioural change is a challenge, as individuals usually regain their original body fat levels after the end of treatment [19, 20]. Ongoing research aims to optimise behaviour change interventions through a novel framework integrating intervention content, exposure (including reach and engagement), and context [21–23]. These elements interact to affect the behaviour through multiple mechanisms, including the components motivation (automatic, reflective), capability (knowledge, skills), and opportunity (social and physical factors outside the individual) [24].

In addition, despite the recommendation to apply an individualised approach [25, 26], few studies have utilised this in clinical practice. Physical activity on prescription (PAP) in the prevention of disease using an individualized approach [27] has been partly implemented into Swedish healthcare, resulting in short- and long-term favourable effects on physical activity [28, 29]. An individualized dietary approach has not been widely implemented in healthcare settings, despite its effectiveness, due to resource prioritisation towards managing malnutrition and diet-related conditions like cancer and diabetes over obesity [30]. Furthermore, a significant limitation is that the physical activity guidelines (FYSS) [27] and dietary recommendations [31] are based on group level, ‘one size fits all’ approach, which lack individual application. Previous research has shown that it is important to consider individual adaptation to medical conditions (e.g. risk profile, symptoms, and comorbidities) as well as to psychosocial factors (e.g. preferences, ‘what matters most’, barriers, facilitators, and readiness to change) [32, 33]. It is also known that individuals require support for behavioural change (e.g. counselling/coaching, group activities, education, nudging) [34]. Another challenge is achieving sustained weight loss whilst preserving muscle mass and metabolic health [35]. A major determinant is the obesogenic environment, shaping physical activity and dietary habits [10, 11]. Participants in weight loss studies often cite environmental barriers to dietary adherence, yet mechanisms linking these factors to weight management remain underexplored [36].

Technological progresses now facilitate more precise measurements of physical activity [37], energy expenditure [38], and food and nutrient intake [39], which paves the way for more tailored interventions. Implementing effective interventions necessitates a multifactorial approach that considers individual and environmental factors [40]. This requirement places new demands on competencies within healthcare and may potentially involve interdisciplinary teams. Evaluating the effectiveness of these interventions necessitates careful monitoring of outcomes. Although individual adaptations to medical conditions and psychosocial factors are already performed in health care, individualisation of support to physical activity and diet behaviour change has rarely been implemented. A paradigm shift towards precision health, akin to the one proposed [41], is required.

### Explanation for the choice of comparator {9b}

To provide a clear evaluation of the efficacy of the intervention, it will be compared to standard treatment. This comparison will allow outcomes to be assessed against a widely accepted standard of care in the current clinical context, serving as a reference point for assessing the additional benefit of the intervention.

### Objectives {10}

The primary objective of this randomised controlled trial will be to identify whether a 6-month individualised lifestyle intervention for physical activity and diet, enabling a portfolio of activities to support behaviour change, is feasible and leads to larger improvements in weight loss, other cardiometabolic disease risk factors, and health-related quality of life compared to regular, written lifestyle advice, in individuals with overweight or obesity. The hypothesis is that the intervention will be superior to regular lifestyle advice. An additional objective of the study is to identify associations between spatiotemporal environmental exposures and behaviour and health outcomes.

## Methods: patient and public involvement, and trial design

### Patient and public involvement {11}

Patients and the public were not involved in the design, conduct, reporting, or dissemination plans of this study.

### Trial design {12}

This study will be a prospective, single centre, open-label, interventional, longitudinal, two-arm and non-blinded randomised controlled trial employing a parallel 1:1 allocation ratio, i.e. ‘equal allocation’ (ClinicalTrials.gov NCT06379802). The schedule of enrolment, interventions, and assessments during the trial according to Standard Protocol Items: Recommendations for Interventional Trials (SPIRIT) is presented in Table 1. An extended version of Table 1 is provided as Supplementary Material.

**Table 1:** Schedule and the key components of the enrolment, interventions, and assessments

	Study period						
Timepoint		T0			T1	T2	T3
	Recruitment and screening	Baseline assessments	Randomisation	Initial meeting	Follow-ups at 1, 3, and 6 months post-study start		
Enrolment							
Eligibility screen							
Screening 1 Request for study participation Per post and QR-code	✓						
Screening 2 By phone and baseline	✓	✓					
Informed consent		✓					
Allocation			✓				
Interventions							
A 6-month, individualised intervention designed to optimise behaviour change, incorporating objective assessments and a comprehensive portfolio of activities, described in manuscript				✓	✓	✓	✓
Assessments							
Anthropometry part I	✓	✓		✓	✓	✓	✓
Tests part 1		✓			✓	✓	✓
Clinical tests 1		✓			✓	✓	✓
Anthropometry part 2							
Tests part 2							
Accelerometer introduction given		✓					✓
Questionnaires		✓					✓
Blood samples		✓					✓
Accelerometer data		✓					✓
Energy		✓					✓
Risk assessment		✓					
Relative contra indications		✓					
Start—initial meeting							
1a). Plan for achieving 300 min of aerobic physical activity (PA) of moderate intensity/w				✓			
1b) Plan for muscle strength training				✓			
2) Plan for diet/calorie restriction and behavioural change				✓			

Timepoints are shown for screening visit, baseline (T0), 1-month follow-up (T1), 3-month follow-up (T2), and 6-month follow-up (T3). The tick mark indicates when each procedure is performed

*BMI* body mass index, *SBP* systolic blood pressure, *DBP* diastolic blood pressure, *P HbA1c* glycated haemoglobin, *SGPALS* The Saltin-Grimby Physical Activity Scale, *MVPA* moderate and vigorous intensity physical activity level, *EQ-5D-3L* EuroQol 5 dimensions, 3 levels, *P in front of blood test*, plasma, *PA* physical activity, *SCORE2* Systematic COronary Risk Evaluation 2 (estimated risk of experiencing a cardiovascular event within the next 10 years)

Follow-up was conducted at one and three months in the intervention group and at six months in both groups afterbaseline

## Methods: participants, interventions, and outcomes

### Trial setting {13}

This trial will be conducted at the Centre for Lifestyle Intervention (CLI) at Sahlgrenska University Hospital/Östra in Gothenburg, Sweden. CLI is a collaborative centre established in partnership between Sahlgrenska University Hospital and the University of Gothenburg.

### Eligibility criteria for participants {14a}

#### Inclusion criteria

Individuals, females and males, of any race, ethnicity and ancestry, aged 45–65 years with a body mass index (BMI) of ≥28 and <35 and living within Gothenburg and its surrounding areas, will be included.

#### Exclusion criteria

Individuals with known coronary artery disease (i.e. clinical symptoms/earlier event) or other contraindications such as anti-obesity medications (such as GLP-1 agonists), language barriers, or unable to perform lifestyle interventions will be excluded.

### Eligibility criteria for sites and those delivering interventions {14b}

Not applicable.

### Who will take informed consent? {32a}

Prior to any evaluations, the research nurse will obtain informed consent from participants, who are required to sign two copies of the informed consent form, retaining one for their records.

### Additional consent provisions for collection and use of participant data and biological specimens

The consent form will include a statement whereby participants agree to the storage of their samples in a biobank as outlined in the participant information sheet. Blood samples will be collected and sent to the Clinical Chemistry laboratory at the Sahlgrenska University Hospital/Östra for immediate analysis and to the Biobank Väst for storage.

## Intervention and comparator

### Intervention and comparator description {15a}

**Fig. 1:**
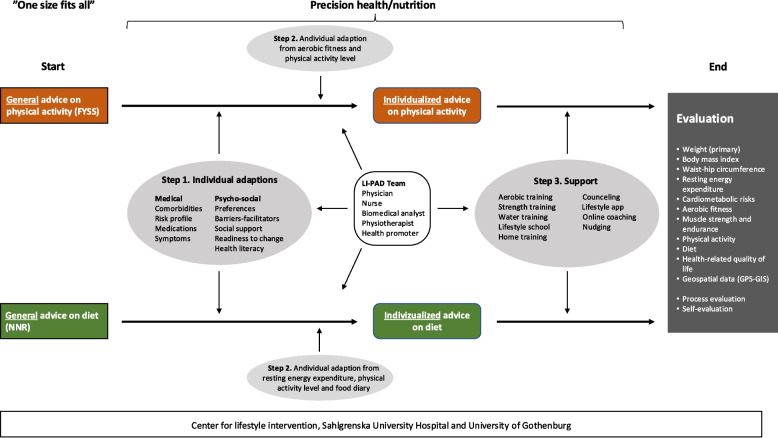
Visual overview of the intervention including the different steps used to aim for precision health

A comprehensive overview of the intervention including the different steps used to aim for precision health is shown in Fig. 1. The behaviour change intervention will be constructed based on current evidence on content, delivery and behaviour change techniques [16, 34, 41–44], to approach precision health [45, 46]. More specifically, individualised advice on physical activity and diet will be provided based on medical risk profile and psychosocial factors, preferences, and objective measures of aerobic fitness, physical activity, and resting energy expenditure. Each participant will be offered a counselling session of 60 min weekly for the first four weeks, succeeded by shorter sessions of 30 min every third week until the end of the intervention, approximately 12 sessions in total. The sessions will support behaviour change towards the individualized advice on physical activity and diet. Multiple counselling sessions are important for successful behaviour change [16]. The counselling sessions will be held at the CLI or online and led by a trained health promoter with a degree in health promotion and food and nutrition sciences. The health promoters that have in-depth knowledge of behavioural change, motivational interviewing, health promotion, and food and nutrition will follow a structured protocol, with a stepwise use of multiple behaviour change techniques to support motivation and capability for self-regulation, such as assessment of motivation and capability, goal setting with action plan, self-monitoring of behaviour and feedback on performance, review/revision of goals/action plan, problem-solving, and stimulus control [16, 43, 45]. In addition, access to lifestyle schools, physiotherapist-led training of aerobic exercise and resistance training, biannual swimming facility pass, and smartphone lifestyle app will be offered, and the participants will also be informed about other tools such as the Lifestyle tool (in Swedish, Livsstilsverktyget) and Health Coach Online (in Swedish, Hälsocoach online).

### Intervention components

#### Initial meeting

The initial consultation will take place at SU/Östra, where participants in the intervention group will meet with a research nurse, physician, or physiotherapist. This session will provide comprehensive feedback on baseline assessment results, including blood pressure measurement, blood samples, and aerobic fitness (evaluated via the submaximal Ekblom-Bak test) [47], and estimated cardiovascular risk score will be derived using the Systematic Coronary Risk Evaluation 2 (SCORE2) algorithm [48]. Furthermore, participants will receive individualised counselling on physical activity, tailored to their aerobic fitness (relative moderate intensity of aerobic activity) and objectively measured physical activity, as assessed by accelerometer data [37]. Resting energy expenditure will be evaluated through indirect calorimetry adhering to evidence-based criteria [49]. Subsequently, participants will engage with a health promoter for further guidance and support in achieving their personalised health goals.

#### Advisory on physical activity

If the baseline fitness level will be low (maximal oxygen uptake (VO_2max_) 20–25 ml/kg) [50], it will be essential to provide advice on activities that promote fitness improvement [51]. The exercise test on the cycle ergometer can serve as an example to demonstrate experiences during moderate-intensity activities, aiming for a perceived exertion of around 12–13 on the Borg scale [52]. Moderate-intensity activities typically result in an increase in heart rate and breathing (Borg scale ratings of 12–13 [50].

The goal will be to meet the general recommendations set by Physical Activity in Prevention and Treatment of Disease [53] and Nordic Nutrition Recommendations [54] with an aim to achieve specific recommendations for individuals with overweight and obesity. To acknowledge the complex interplay between individual fitness and the relative intensity of physical activity, the personalised advice provided will be tailored to the fitness level of each participant in relation to their absolute physical activity intensity based on accelerometry data (Additional file 1), as illustrated in previous research [37].

#### Advisory on diet

Based on measured resting energy expenditure (REE), using indirect calorimetry, physical activity level (PAL), and accelerometry at baseline, the individual energy expenditure (EE) will be assessed. To promote a weight reduction of 5–10% at the end of the 6-month intervention period [25], an energy deficit of 500 kcal/day will initially be applied, aimed at the target weight loss [38], as advised for weight reduction [25]. An additional 200 kcal/day energy deficit may be applied if slower weight reduction than the expected 0.5 kg/w is observed at the 1-month and 3-month follow-ups [25], provided that the participant has been compliant to the prescribed energy intake.

Self-monitoring of physical activity and diet behaviour will be performed using the CLI lifestyle app (detailed description of the app below). Based on the advised EI, individual daily meal plans will be suggested providing adequate intake of macro- and micronutrients according to Nordic Nutrition Recommendations (NNR) [54]. These meal plans are modifications from initial recordings by the participants in the CLI app for pedagogical purposes. Pedagogical aids provided by the Swedish Food Agency such as the food circle and the plate model will be used to enhance understanding.

#### Behaviour counselling sessions

The aim of the counselling sessions will be to support motivation and capability (knowledge, skills) for physical activity and diet behaviour change to reduce body weight, using evidence-based self-regulation behaviour change techniques [40, 42]. Each participant will be offered a counselling session of 60 min every week for the first 4 weeks, succeeded by shorter sessions of 30 min every third week until the end of intervention, approximately 12 sessions in total. The counselling sessions will be held at the hospital or online and led by the health promoter. They will follow a structured protocol, with a stepwise use of the behaviour change techniques assessment of motivation and capability, specific goal setting with action plan, self-monitoring of behaviour using the CLI app, feedback on performance, review/revision of goals/action plan, problem-solving (barriers and facilitators), and stimulus control (objects, activities, experiences, or emotions provoking a behaviour). In the counselling sessions, the participant will receive individualized advice and support for physical activity and diet behaviour change based on medical and psychosocial factors, preferences, and objective measures of aerobic fitness, physical activity, and resting energy expenditure presented above.

#### Lifestyle school

The Lifestyle School will comprise four themes and a movie. Each thematic session will last 2 h, whilst the movie will be run for just under 30 min. The movie will be accessible at any time via the smartphone application. The remaining components of the Lifestyle School will be led by a health promoter and will be conducted as a group activity on a triweekly basis, rotating through the four themes. The Lifestyle School is described in detail in Additional file 1.

#### Physiotherapist-led training of aerobic exercise and resistance training

Participants in the intervention group will be offered aerobic physical activity training and resistance training at the Department of Physiotherapy, Sahlgrenska University Hospital. Each training component will be conducted for 1 h, twice weekly.

Aerobic training sessions will be structured as group activities, typically involving multiple participants, conducted under the supervision of a physiotherapist. To maintain intervention consistency, sessions will proceed as planned even when attendance is limited to a single participant. In these cases, the physiotherapist will conduct the session on a one-to-one basis, adapting the planned group format to suit individual delivery. To promote adherence to moderate-intensity aerobic training, a dedicated element will be conducted. During the first session, participants’ perceived exertion levels will be evaluated using the Borg Rating of Perceived Exertion (RPE) Scale [52]. Concurrently, heart rates will be monitored via pulse oximetry. Participants will be instructed to maintain an intensity level corresponding to 12–13 on the Borg scale, which is indicative of moderate exertion.

The resistance training will incorporate a diverse range of equipment, including machines, free weights and bodyweight exercises, and is conducted under the supervision of a registered physiotherapist. The strength training component will follow a different approach, with each participant assigned a bespoke programme tailored to their specific needs and capabilities. The individualised sessions will be conducted in a versatile training facility at the Physiotherapy Department, Sahlgrenska University Hospital/Östra. This well-equipped space will accommodate varying participant numbers, facilitating both solitary and concurrent training sessions.

#### Precision health

The present study will mark an advancement in lifestyle intervention programmes by transitioning from traditional randomised controlled trials to embrace precision health [45, 46] (see Fig. 1). This will be achieved through:

1. Personalisation of the intervention to account for medical conditions, personal and environmental factors, participant preferences, and psychosocial factors.
2. Tailored physical activity (PA) and dietary advice, with individual adaptations, based on data from food diaries, motivational interviewing, and objective measures of resting energy expenditure, aerobic fitness (predicted VO_2max_), and physical activity based on accelerometery data.
3. Supportive tools to help participants achieve behavioural change, including educational resources, skills training, and ongoing support.

#### Standard care

The control group will receive standard care, defined as written basic lifestyle advice. This advice will be based on general recommendations for physical activity and diet, following a ‘one-size-fits-all’ approach. These recommendations will be derived from the World Health Organization guidelines on physical activity and sedentary behaviour, 2020 [55], and from the NNR 2012 [54]. For weight loss, these recommendations will encompass a minimum of 300 min per week of moderate-intensity aerobic physical activity, resistance training twice weekly, and a nutritionally balanced, varied diet. Moderate-intensity physical activity will be selected to ensure sufficient intensity for weight loss and improvement in the biomarkers of interest, whilst not compromising medical safety. NNR 2012 dietary guidelines will advocate for a nutritional pattern characterised by a high intake of plant-based foods, including vegetables, legumes, fruit, berries, complemented by regular consumption of fish, shellfish, nuts and seeds, and a limited consumption of red and processed meat, salt, sugar, and alcohol. NNR 2012 [54] will further recommend a switch to wholegrain in i.e. pasta, bread, grain, and rice, healthy fats, such as vegetable liquid fats made from, e.g. rapeseed oil, and healthy sandwich spreads, and low-fat dairy products [54]. Following randomisation to the control group, participants will be contacted via telephone by a health promoter or a study nurse, who will inform them of their allocation and guide them to the CLI lifestyle app, a specially designed mobile app for LI-PAD, through which they will be able to have access to the recommendations.

#### The participant interface—CLI Lifestyle application

The smartphone application ‘Livsstilsapp’ (CLI Lifestyle app) will be accessible through smartphones and tablets. Within this app, participants in the control group will have access to the general recommendations for physical activity and diet. Conversely, those in the intervention group will receive enhanced resources and functionalities in the app, which include the ability to schedule training sessions with one of three physiotherapists, a basic strength training programme and a cardio exercise regimen. Additionally, the app will feature comprehensive software for tracking food intake, developing dietary plans, and providing nutritional insights, with feedback integrated from national food and nutrition databases (Nutrition Data Sweden AB, Sweden). Users will also be able to enter self-selected body measurements, such as weight, waist circumference, and hip circumference.

#### Environmental exposures

Furthermore, participants will also be offered to enable Global Positioning System (GPS) tracking within the application. Using GPS data, individual environmental exposures along GPS routes during the 6-month study period will be identified. Given the lack of standard for optimal environmental measures [56], multiple measures of exposures will be calculated, e.g. density of food outlets (e.g. restaurants and grocery stores) and facilities for physical activity (e.g. gyms, bike paths, sports halls), ratio of healthy/unhealthy food outlets, and temporal exposure along individuals' GPS routes. Association between the environment and adherence to the intervention, health behaviours, and outcomes will be explored. This will also allow objective measurement of physical activity levels in both the intervention and control groups [57].

### Criteria for discontinuing or modifying allocated interventions {15b}

Participants may be discontinued from the study based on two primary criteria: firstly, at their own request and/or secondly, due to a deterioration in health status (of any reason) that renders them unable to continue the intervention. Anti-obesity medications (e.g. GLP-1 drugs) that could interfere with the study outcomes will constitute an exclusion criterion. We will record any newly prescribed medications during the study.

### Strategies to improve adherence to interventions {15c}

The individualized advice based on objective measures and individual preferences, support from a health promoter and a physiotherapist, the opportunity to choose between in-person and online meetings, and reminders for scheduled appointments will aim to contribute to improved adherence to interventions.

### Concomitant care and interventions that are permitted or prohibited during the trial {15d}

Anti-obesity medications (e.g. GLP-1 drugs) that could interfere with the study outcomes will constitute an exclusion criterion. We will record any newly prescribed medications during the study.

### Ancillary and post-trial care {34}

Participants in the study will be covered by the patient injury insurance of the region.

### Outcomes {16}

#### Primary outcomes

The primary outcome will be weight loss in (%) from baseline to 6 months. Additional primary outcomes will include weight loss from baseline to 1-month follow-up, from 1-month follow-up to 3-month follow-up, and from 3-month follow-up to 6-month follow-up.

#### Secondary outcomes

The secondary outcomes will incorporate changes in cardiometabolic risk factors, including weight, BMI, waist and hip circumferences, and systolic and diastolic blood pressure (SBP and DBP) that will be assessed at intervals described for weight loss. Additionally, assessed at baseline and the 6-month follow-up, secondary outcomes will also include changes in (1) liver status via plasma aspartate aminotransferase (P-ASAT) and plasma alanine aminotransferase (P-ALAT); (2) metabolism via P glucose, blood (B), haemoglobin A1c (HbA1c) and P insulin; and (3) lipid status via P cholesterol, low-density lipoprotein cholesterol (LDL-C) and high-density lipoprotein cholesterol (HDL-C), and triglycerides. All blood samples will be taken in a fasting state. Change in aerobic fitness will be measured by predicted VO_2max_, and changes in physical activity will be assessed using the Axivity AX3 accelerometer (Axivity Ltd, UK) [37], self-reported physical activity levels will be assessed via the Saltin-Grimby Physical Activity Level Scale (SGPALS) [58], and changes in muscle strength and endurance will be measured by handgrip strength, sit-to-stand, shoulder flexion, and toe rise tests and will be based on assessments at baseline and the 6-month follow-up. Changes from baseline to 6-month follow-up will also include dietary patterns and nutrient intake, using the Meal-Q food frequency questionnaire [59] and resting energy expenditure using indirect calorimetry (COSMED Q-NRG, COSMED Srl, Rome, Italy). Resting energy expenditure will also be measured between the following intervals: baseline to 1-month follow-up, 1-month to 3-month follow-up, and 3-month to 6-month follow-up.

Changes in health-related quality of life (HRQoL) [59] will be assessed from baseline to the 6-month follow-up using the patient-reported outcome measure (PROM), specifically the EuroQol 5-dimension, 3-level scale (EQ-5D-3L) [60] developed by the EuroQol Group [60]. The results will be presented descriptively as distributions of participants across EQ-5D-3L dimensions and health state profiles. For each of the five dimensions (mobility, self-care, usual activities, pain/discomfort, and anxiety and depression), the number and percentage of participants in each of the three levels (1 = no problems, 2 = some problems, and 3 = extreme problems) will be reported. The health state profiles will be based on the five digits, e.g. 11111 for full health and 33333 for worst health. All outcomes will comprehensively be described in Additional file 1.

### Harms {17}

All adverse events will be recorded and registered.

### Participant timeline {18}

**Fig. 2:**
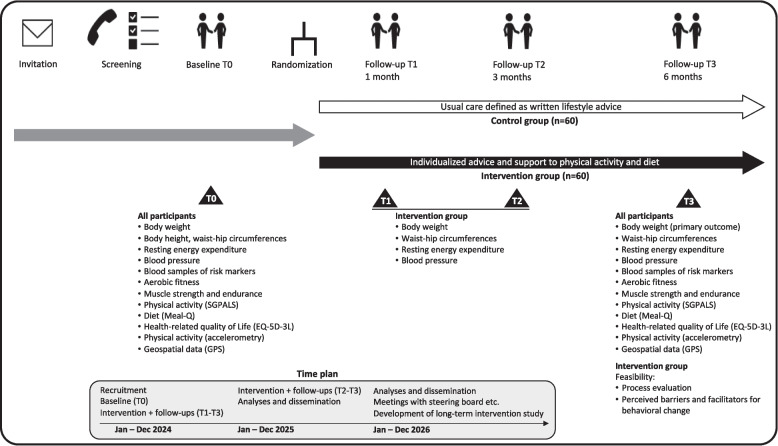
Overview of the study timeline, key components, and milestones

A visual overview of the study timeline, key components, and milestones, facilitating quick comprehension, is shown in Fig. 2. A thorough exposition of the methodological approach is presented in Additional file 1.

### Sample size {19}

To detect a mean weight reduction of 5% in the intervention group compared to the control group (effect size) with 80% power, at least 60 participants will be required in each group. The equation for calculating the sample size in each group to detect a specific effect size (*d*) will be given by:

$$n=2\times\left(Za+Z\left(1-\beta\right)\right)\hat{\phantom{0}}2\times\left(\mathrm{SD}/d\right)\hat{\phantom{0}}2$$

where *Zα* is 1.96 for *α* = 0.05 and *Z*(1 − *β*) is 0.85 for *β* = 0.2 (i.e. 80% power), with the standard deviation (SD) set at 10% and the effect size (*d*) of 5% [60]. The SD value is derived from the Look AHEAD study [61].

### Recruitment {20}

Potential participants will be randomly recruited from the population within the Greater Gothenburg metropolitan area. Invitations will be sent via mail. To obtain relevant demographic information, the Swedish State Personal Address Register (SPAR) [62] will be utilised. A study invitation, along with detailed information, will be sent to 1000 individuals at a time and distributed in batches until 120 individuals have been enrolled. Those who will believe they fit the criteria of having a BMI between >28 and <35 and want to lose weight will be encouraged to contact Centre for Lifestyle Intervention by email, phone, or the national web-based platform (1177.se) (a website that provides extensive health-related information, self-care advice, and e-services).

## Assignment of interventions: allocation

### Sequence generation: who will generate the sequence {21a}

Based on the a priori power calculation, a total of 120 participants will be randomly assigned in a 1:1 ratio to either the intervention group or the control group. The allocation sequence will be generated manually by a research nurse.

### Sequence generation: type of randomisation {21b}

Simple randomisation without stratification or blocking will be applied, with an allocation ratio of 1:1. To ensure equal group sizes (60 participants per group), a fixed number of allocation slips will be prepared in advance. No additional restrictions such as blocking or stratification will be used.

### Allocation concealment mechanism {22}

Allocation concealment will be ensured using opaque envelopes prepared in advance by the study coordinator. Two envelopes labelled ‘Intervention’ and ‘Control’ will each contain 60 pre-marked allocation slips. During the randomisation procedure, a research nurse will draw allocation slips blindly and without prior knowledge of their content. The selected slip will be transferred to an envelope labelled ‘Completed’ together with the participant’s study ID to ensure traceability and prevent reuse. The envelopes will be stored securely and accessed only at the time of randomisation.

### Implementation {23}

Randomisation will be conducted 1–2 times per week by the study coordinator in collaboration with one of the research nurses. Participants will be randomised in chronological order according to completion of their baseline assessments. Baseline assessments will be performed in batches of 3–4 week periods, with scheduling adjusted to accommodate a planned 4-week holiday period. Following randomisation to the intervention group, participants will be invited to attend an initial briefing session.

## Assignment of interventions: blinding

### Who will be blinded? {24a}

Randomisation will be conducted randomly and in a blinded manner, but no further blinding will be implemented.

### How will blinding be achieved {24b}

Not applicable.

### Procedure for unblinding if needed {24c}

Not applicable.

## Data collection and management

### Plans for assessment and collection of outcomes {25a}

#### Pre-screening

For those who will be interested in participating in the study, an initial screening will be conducted over the phone by one of two research nurses. During this screening, the potential participants will be asked about their age, sex, weight, height, and whether their BMI falls within the predetermined range. The research nurse will also ensure that the person has sufficient proficiency in Swedish to understand and communicate effectively. Additionally, the research nurse will be inquire about any history of myocardial infarction, stroke, transient ischaemic attack, current or previous angina pectoris, or other relevant heart conditions (e.g., arrhythmia or aortic stenosis), as well as other medical conditions that could impact the ability of the participant to engage in the study (e.g., severe multiple sclerosis, end-stage AIDS, metastatic cancer, or systemic diseases). Those who will meet the predefined inclusion criteria will be invited to undergo baseline assessments.

#### Clinical assessments

Beyond the two primary clinical visits—baseline (T0) and 6-month follow-up (T3)—participants will be assigned to the intervention group to undergo additional evaluations at 1 month (T1) and 3 months (T2) post-study initiation.

#### Baseline assessment

Baseline assessments will be conducted on-site at the Centre for Lifestyle Intervention at Sahlgrenska University Hospital/Östra (SU/ÖS) in Gothenburg by one of two trained research nurses. The process will begin with participant identification verification, followed by a thorough review of adherence to pre-assessment instructions. These instructions will include fasting for a minimum of 4–5 h, abstaining from food, alcohol, tobacco products, coffee, and caffeine for the preceding 4 h, and refraining from moderate physical activity for 2 h or vigorous exercise for 14 h prior to assessment. The comprehensive baseline assessment protocol will encompass personal data collection, anthropometric measurements, resting energy expenditure quantification, blood pressure monitoring, blood sampling and fitness evaluations, and muscle function tests. Additionally, participants will be introduced to the Axivity AX3 accelerometer for objective physical activity measurement. For detailed information about the assessments, see Additional file 1.

#### Risk assessment

The baseline assessment results, including the blood samples aspartate aminotransferase (ASAT), alanine aminotransferase (ALAT), plasma glucose, plasma insulin, glycated haemoglobin (HbA1C), total cholesterol, low-density lipoprotein cholesterol (LDL-C), high-density lipoprotein cholesterol (HDL-C), and triglycerides, will undergo a comprehensive review by one of two study physicians as part of a rigorous risk assessment process. In addition to previously described blood sample analyses, C-reactive protein will be used to exclude those with ongoing infection. This evaluation will determine the participants’ medical suitability for study inclusion. The risk assessment components are delineated in Fig. 1. This systematic approach will ensure that only medically appropriate candidates proceed to the intervention phase. Participants meeting the medical eligibility criteria will be subsequently randomised to either the intervention or control group. The blood samples will also be used to evaluate changes in cardiometabolic risk factors from baseline to the 6-month follow-up.

#### 1-month follow-up (intervention group)

Participants in the intervention group will undergo a 1-month follow-up assessment at CLI, SU/Östra. This comprehensive evaluation, conducted by a health promoter or research nurse, will encompass measurements of body weight, waist-to-hip circumference, systolic and diastolic blood pressure (SBP and DBP), and resting energy expenditure.

#### 3-month follow-up (intervention group)

At the 3-month follow-up, also conducted at CLI, SU/ÖS for the intervention group, a research nurse will perform a comprehensive assessment including body weight, waist-to-hip circumference, SBP and DBP, and resting energy expenditure measurements, following the same procedure as in the 1-month follow-up. The 1- and 3-month follow-up evaluations will be performed to enable adjustments to individual treatment regimens.

#### 6-month follow-up (all participants)

The 6-month follow-up, held at CLI, SU/ÖS for all participants, will replicate the baseline evaluations in scope and content. Additionally, this assessment will incorporate an evaluation of the Advisory Sessions and the Lifestyle School program. It will serve as a critical stage for assessing the efficacy of the intervention and comparing outcomes between the intervention and control groups. The follow-up will include written summarised feedback that encompasses weight, weight change from baseline and the 6-month follow-up, BMI, waist circumference, and blood test results (blood glucose, blood lipids [total cholesterol, LDL-C, and HDL-C] [39], systolic and diastolic blood pressure, and fitness value from the Ekblom-Bak test at baseline and the 6-month follow-up. Using a web-based questionnaire, Research Electronic Data Capture (REDCap), the intervention group will undertake a self-assessment regarding their adherence to dietary and physical activity (aerobic and strength) recommendations. Participants will self-assess whether they have met these guidelines and indicate their intentions to either maintain current behaviours, increase efforts to achieve goals, or forgo pursuing the objectives. In addition, they will be asked to outline their strategy for reaching the goal. Furthermore, the intervention group will be prompted to evaluate their satisfaction with any weight changes experienced. They will be asked whether they intend to pursue further modifications to achieve their target or if they are content with their current weight status. The participants will also be asked whether they are satisfied with their weight change and if they will continue with weight change to reach the goal or not. For more detailed information, see Additional file 1.

#### Final report

Following the completion of the 6-month follow-up, all participants will receive a comprehensive medical report by mail. This report, authorised by the study physician, will present a detailed comparison of data at baseline and at the 6-month follow-up. The data will include weight, percentage weight change, BMI, waist circumference, blood samples (glucose, S-cholesterol, LDL-C, HDL-C), SBP and DBP, and fitness value (considered age and sex). This report will enable a thorough examination of changes over time and includes a brief comment from the physician.

### Plans to promote participant retention and complete follow-up {25b}

To enhance participant retention and ensure comprehensive follow-up, the study protocol will incorporate several strategies. Participants will be sent reminder text messages to maintain engagement. To facilitate adherence to the intervention, the study will offer the option of virtual consultations (conducted online) as an alternative to in-person appointments. The individualisation itself (advice based on objective measures and individual preferences) will also contribute to the participants completing the follow-up.

### Data management {26}

The study will be conducted in a consistent and controlled manner, adhering to the research protocol through the implementation of standard operating procedures (SOP), which will ensure standardised processes and compliance with regulatory and quality requirements. All study data will be entered and managed using REDCap, a web application that will enable the creation and management of data, hosted at the University of Gothenburg. Data entry will be performed by trained research personnel with built-in range checks and validation rules to minimise errors. Applying standardised data collection, trained research personnel and employing control group and REDCap, will reduce the risk of measurement bias. The use of clear data standards in the REDCap and predefined statistical methods will reduce misinformation bias. Accelerometer and GPS data will be processed using standardised protocols and stored on secure servers with unique participant identifiers. All electronic data will be stored on encrypted, password-protected servers with access restricted to authorised study personnel. Regular data backups will be performed daily. Offsite backups will be conducted weekly. Paper documentation will be stored in locked cabinets within secure facilities at the CLI. Artificial intelligence tools will be applied to optimise word choice only in situations where linguistic uncertainty arises due to our status as non-native English speakers.

### Confidentiality {33}

Personal information will be protected in accordance with the European General Data Protection Regulation (GDPR) and Swedish data protection laws. Each participant will assign a unique study identification number used on all study documents, specimens, and in the database. The link between identities and study IDs will be maintained in a separate, encrypted database accessible only to designated research staff. GPS data will only be presented at an aggregated level for environmental exposure research. Data transfers will occur via secure, encrypted channels, and data that will be shared for analysis will be de-identified.

## Statistical methods

### Statistical methods for primary and secondary outcomes {27a}

To accommodate the diverse nature of the variables, both linear and logistic analyses will be employed for continuous and categorical variables, primary and secondary outcomes included. To evaluate variation at both group and individual levels, multilevel mixed modelling for repeated measures will be implemented, providing a comprehensive assessment of intervention effects across multiple time points, adjusted for baseline values, age, and sex. In addition, if there are substantial differences in other values, appropriate adjustments will be made. The complexity of highly collinear variables, such as physical activity intensity levels, will be addressed using partial least squares (PLS) regression. Further statistical analyses may incorporate structural equation modelling, enabling the identification of interrelationships among intervention components and outcome measures without necessitating partial least squares regression for repeated measurements. Both intention-to-treat (primary) and per-protocol analyses (secondary) will be conducted to assess treatment efficacy. Also, machine learning, spatiotemporal regression models, and functional regression analysis will be applied.

### Who will be included in each analysis {27b}

To investigate heterogeneity of intervention effects and identify potential moderators of treatment response, several pre-planned subgroup analyses will be conducted using the same statistical framework as the primary analyses. Subgroups will be examined based on protocol adherence levels, demographic characteristics (age and sex), baseline BMI categories, and baseline fitness levels (stratified as low or moderate to high based on predicted VO_2max_).

Within the intervention group, comparative analyses will evaluate differences in outcomes among participants who engaged with different support components (behaviour counselling sessions, lifestyle school, physiotherapist-led training, etc.). Responder analyses will compare participants who achieved the target weight reduction goal (≥5%) with those who did not, investigating potential predictors of successful weight loss. Additionally, analyses of participants with decreased versus maintained resting metabolic rate will explore the relationship between changes in energy expenditure and weight management outcomes.

All subgroup analyses will be clearly identified as exploratory in nature, with findings interpreted cautiously in the context of biological plausibility and consistency with established evidence.

### How missing data will be handled in the analysis {27c}

Mixed-effects models will facilitate handling missing data.

### Methods for additional analyses (e.g. subgroup analyses) {27d}

Intention-to-treat (ITT) analysis, by including all participants in their allocated groups regardless of adherence, will be the primary approach. To assess efficacy under ideal conditions, restricted to participants who adhered to the intervention protocol, per-protocol (PP) analysis will be the secondary method.

### Interim analyses {28b}

To maintain the integrity of the study and avoid potential pitfalls, we will not perform interim analyses.

### Protocol and statistical analysis plan {5}

The LI-PAD protocol and statistical analysis plan has been published on ResearchWeb and ClinicalTrials.gov and is also available in the relevant publication. According to Swedish legislature, we do not have permission to share participant-level data with others or to present data in a manner that could lead to the identification of individual participants. Programming languages that will be used for statistical code include R and Matlab.

## Oversight and monitoring

### Composition of the coordinating centre and trial steering committee {3d}

The coordinating centre consists of a multidisciplinary team with expertise in clinical research, data management, and health promotion. The research team includes a principal investigator and two senior researchers including a coordinator/clinical research leader who will oversee the trial. In addition, the team comprises a data specialist ensuring data quality and a nutritionist providing expertise input on dietary aspects, as well as two research nurses and two health promoters. Thereto, data will be collected in a digital electronic case report form (REDCap) with predefined ranges and automated alerts, thereby facilitating efficient and enhancing patient safety and data quality. To support the trial, the project also collaborates with the Department of Physiotherapy, Sahlgrenska University Hospital/Östra. The steering committee that consists of the principal investigator, one representative from the University of Gothenburg, one representative from Sahlgrenska University Hospital, and an adjunct professor is responsible for the overall research strategy and vision.

### Composition of the data monitoring committee, its role and reporting structure {28a}

This study will not include a formal Data Monitoring Committee (DMC). It is based on the low-risk nature of the intervention (and the relatively small scale of the trial). Instead of a formal data monitoring committee, patient safety and data quality will be monitored by a structure led by the principal investigator and two senior researchers, including the coordinator/clinical research leader. All adverse events will be recorded and registered by a data specialist.

### Frequency and plans for auditing trial conduct {29}

We will not conduct any trial auditing as no pharmaceutical interventions will be included.

### Protocol amendments {31}

Any deviations from our standard operating procedure (SOP) will be reported to the data manager at CLI. If we make substantial amendments, they are communicated to ClinicalTrials.gov and the Swedish Ethical Review Authority. Amendments may necessitate the submission of a formal Amendment Application to the Swedish Ethical Review.

### Dissemination policy {8}

The dissemination plan includes presenting findings at academic conferences, publishing results in high-impact, peer-reviewed journals, and presenting findings at meetings at Sahlgrenska University Hospital and the University of Gothenburg, and on our website, at Sahlgrenska liv (newsletter at SU), Akademiliv (newsletter at GU), and in infographics for social media dissemination. We will also engage with policymakers by organising workshops to explore the implications of the findings.

## Discussion

### General discussion

This study will evaluate the efficacy of a 6-month intervention approach that individually optimises lifestyle behaviours, specifically related to physical activity and diet. It will aim to determine whether this personalised strategy, in accordance with the ‘precision health’ paradigm previously proposed [41], will lead to more substantial improvements in body weight, cardiometabolic risk factors, and health-related quality of life compared to standard written lifestyle advice in individuals with overweight or obesity. By integrating objective measures—including resting energy expenditure, aerobic fitness, and physical activity—into tailored interventions in physical activity and diet, the intervention is designed to optimise health outcomes. The intervention content and delivery are based on current evidence for the most effective behaviour change intervention with physical activity and diet [43, 44]. The findings will provide valuable insights into the implementation of individualised care approaches in clinical settings, potentially informing future precision health strategies for obesity management and cardiometabolic risk factor reduction.

Although we will include objective measures of physical activity, aerobic fitness and resting energy expenditure to approach precision health for individualised physical activity and diet prescription, additional steps will be required to truly reach precision at the individual level. We will employ a pragmatic alternative feasible in clinical practice to determine energy need for planning energy intake promoting weight reduction of 5–10% at a 6-month intervention, using accelerometer and resting energy expenditure data with a common interval of energy deficit of 500–750 kcal/day [25]. The most accurate and precise assessment of energy need under free-living conditions is the doubly labelled water (DLW) method [63]. Although DLW has developed to become a more accessible method to determine energy need in research and technological advancements have reduced the cost of analysis, it is still too costly to be used in most clinical practice.

Further, whilst objective measures of physical activity and energy expenditure have existed for several decades, it is only recently that objective measures of food intake have become available [39]. Multiple biomarkers have been identified related to the intake of specific foods and food components such as whole grain, fruit and vegetables, red meat, and fish [39]. However, their utility for the assessment of food intake in individuals needs further validation and refinement [39]. In addition, dietary intake is complex as it encompasses several foods and interacting nutrients. Consequently, objective measures of physical activity, energy expenditure, and dietary behaviours to reach precision health will require further development for more precise obesity management.

Finally, behaviour change interventions have undergone major reconstructions to standardise key elements, which include behaviour change techniques, content and delivery, and their relation to the core mechanisms for behaviour change, i.e. motivation, capability, and opportunity [40, 42]. The purpose will be to facilitate future design and planning of behaviour interventions as well as to improve their effectiveness. Consequently, the evidence for selecting the most optimal behaviour change intervention content and delivery is not yet sufficient to contribute to precision health in clinical practice. Thus, there is a need to study and arise more individualised multidimensional intervention programmes.

### Expected outcomes and impact

We hypothesise that the intervention will demonstrate superiority, yielding clinically significant improvements in body weight, physical activity, diet, and cardiometabolic outcomes compared to the control group. By delivering individualised, objectively grounded advice, reinforced through dietary tracking and sustained support from health promoters and physiotherapists, this approach is expected to enhance participant engagement and short-term lifestyle modifications. The multifaceted nature of the intervention, combining personalised counselling with objective measurements and ongoing support, is designed to address the complex challenges of behavioural change in the management of obesity and the risk of cardiometabolic disease. We anticipate that this comprehensive strategy will lead to more substantial and sustained improvements in health outcomes compared to standard care. The results of this study will inform the enhancement of best practices in primary prevention, elucidating actionable strategies for healthcare systems to support health benefits and potentially alleviate future strain on resources.

### Clinical relevance

This intervention is expected to have significant relevance for clinical practice (primary prevention). Evidence-based methods to increase behavioural change in patients with overweight are needed. Such methods will likely include several components and be more individualised. By targeting critical health metrics such as body weight, blood pressure, physical activity and diet—key factors in cardiometabolic risk management—the study will inform evidence-based strategies for delivering personalised lifestyle interventions taking steps towards precision health. The clinical relevance of this initiative will be accentuated by the transformative challenges facing contemporary healthcare systems, tackling the increasing need for preventive treatments. With mounting financial pressures and an imperative for patient-centred efficiency, healthcare will need to prioritise interventions with demonstrable clinical efficacy and with the potential for lasting impact. Improved physical activity and diet behaviours are estimated to have large cost benefits for the health care and society [64]. Considering the critical issue of intervention scalability amidst healthcare workforce shortages across many countries, the current study will concurrently provide valuable insights into the components associated with successful outcomes. These insights might, in turn, help to inform the future development of interventions that could be feasibly implemented within healthcare settings constrained by limited resources.

### Innovations

The introduction of professional health promoters in a clinical setting and the provision of individualised advice, based on objective measures (VO_2max_, REE, and PAL), will represent a novel step compared with the existing healthcare personnel and traditional methods of patient care.

### Strengths

By combining objective measures of physical activity, aerobic fitness, and resting energy expenditure, the study will aim to enhance the individualisation of physical activity and energy intake estimates, thereby informing more precise physical activity and diet behaviour change intervention for obesity management in clinical practice.

### Limitations

This study will acknowledge several limitations. The reliance on self-reported dietary data may introduce recall bias and potentially compromise the accuracy of nutritional assessments. The absence of blinding may lead to assessor bias, potentially influencing outcome measurements. When limited to a 6-month observational timeframe, the assessment of long-term effects will present significant challenges. In addition, variations in participants’ digital literacy may present a barrier to engagement with app-based interventions, potentially affecting adherence and outcomes. Finally, we acknowledge that the generalisability of the study will be limited by its specific population, as participants are restricted to adults aged 45–65 years with a body mass index (BMI) of ≥28 and <35 residing in Gothenburg with sufficient proficiency in Swedish.

### Future directions

Future research should examine the long-term sustainability of interventions beyond the initial study period, focusing on maintenance of behaviour changes and persistence of health improvements. Additionally, adding an investigation of biomarkers of energy need and food intake together with genetic factors and their interaction could enable further personalisation of interventions towards more advanced precision health, potentially enhancing efficacy. Even if successful, economic evaluations are necessary prior to implementation into routine healthcare practice. Comprehensive cost-effective analyses, considering both healthcare and societal perspectives, are crucial to assess economic viability and inform decisions regarding broader implementation. These analyses should account for potential long-term cost savings from prevented or delayed onset of chronic diseases.

## Trial status

This protocol is version 1, dated 12 February 2024. The study commenced on 12 February 2024. The last participant completed the final study vision on 8 May 2025, and the final accelerometer data were received on 9 June 2025. A total of 120 participants were enrolled. Data cleaning and analysis are ongoing.

## Supplementary Information

Additional file 1.

Additional file 2.

## Data Availability

The datasets generated and/or analysed in this study are not publicly accessible in compliance with Swedish regulations. Access to the data may be granted upon request, contingent upon application to and approval by the relevant ethics. Permission to use data can be obtained after an application to and approval by the committee.

## Abbreviations

ALAT: Alanine aminotransferase
ASAT: Aspartate aminotransferase
BMI: Body mass index
CLI: Centre for Lifestyle Intervention
DBP: Diastolic blood pressure
EE: Energy expenditure
EI: Energy intake
EQ-5D-3L: EuroQol 5 dimensions, 3 levels
EQ-VAS: EuroQol visual analogue scale
FYSS: Physical Activity in the Prevention and Treatment of Disease
HbA1c: Glycated haemoglobin (average blood glucose level)
HDL-C: High-density protein cholesterol
HRQoL: Health-related quality of life
LDL-C: Low-density protein cholesterol
LI-PAD: Lifestyle intervention with physical activity and diet
MET: Metabolic equivalent
MVPA: Moderate to vigorous intensity physical activity
NNR: The Nordic Nutrition Recommendations
PA: Physical activity
PAL: Physical activity level
REE: Resting energy expenditure
RQ: Respiratory quotient
SBP: Systolic blood pressure
SGPALS: The Saltin-Grimby Physical Activity Level Scale
SPIRIT: Standard Protocol Items: Recommendations for Interventional Trials
T0: Baseline assessment
T1: 1-Month follow-up
T2: 3-Month follow-up
T3: 6-Month follow-up
TEE: Total energy expenditure
VO_2max_: Maximal oxygen uptake
