# Supplementary material for: An individualised Lifestyle Intervention with Physical Activity and Diet in individuals with overweight and obesity (LI-PAD)—study protocol of a 6-month randomised controlled study {1a}
Source: Trials. 2026 Mar 16;27:232. doi: 10.1186/s13063-026-09606-6 (PMC13019878; doi:10.1186/s13063-026-09606-6)
Supplement: Supplementary file 1 — Additional file 1. [file 13063_2026_9606_MOESM1_ESM.pdf]

# Additional file 1

## PRIMARY OUTCOME MEASURES

### Change in Body Weight - Weight Loss

The primary endpoint will be weight loss in percent change from baseline (T0) to the 6-month follow-up (T3) (all participants), and from baseline (T0) to 1-month (T1) (intervention group only), from 1-month (T1) to 3 months (T2) (intervention group only), and from 3 (T2) months to 6 months (T3) (intervention group only). For all time points, a decrease in body weight will be considered a better outcome than weight gain or weight maintenance.

## SECONDARY OUTCOME MEAUSURES

### Change in Other Cardiovascular Risk Factors

### Body Mass Index (BMI)

Body Mass Index (BMI) (kg/$m^2$), evaluated from baseline (T0) to 6-month follow-up (T3) (all participants), is automatically calculated in Research Electronic Database Capture (REDCap) using the formula BMI = weight (kg)/height$^2$ (m). A BMI of less than 18.5 is classified as underweight, a BMI of 18.5-24.9 as normal weight, a BMI of 25.0 or greater as overweight, and a BMI of 30.0 or greater as obesity.

### Body Weight and Height Measurement

Body weight (kg) is measured using a calibrated weighing scale (Lidén Weighing, Sweden) to the nearest 0.1 kg. Body height (m) is measured to the nearest 0.1 cm using a calibrated

stadiometer (Seca GmbH, Germany). The measurement protocol adheres to standardized procedures. Participants will be instructed to remove footwear and any bulky outer clothing. Any headgear or hairstyles that may interfere with the measurement will be adjusted or removed. The participant stands upright on the stadiometer platform with legs together, heels against the back plate, buttocks, and scapulae in contact with the vertical surface of the stadiometer, arms hanging freely by the sides. The participant is instructed to inhale deeply and maintain the upright posture while the movable headboard is lowered to make firm contact with the vertex of the skull.

## Waist-to-Hip Ratio

Waist-to-hip-ratios are assessed at the following time intervals: from baseline (T0) to 6-month follow-up (T3) (all participants), from baseline (T0) to 1 month (T1) (intervention group), from 1 month (T1) to 3 months (T2) (intervention group), and from 3 months (T2) to 6 months (T3) (intervention group). The waist-to-hip ratio (WHR) is automatically computed in the REDCap database upon entry of waist and hip circumference measurements. The ratio is calculated as: WHR= Waist Circumference (cm)/Hip Circumference (cm).

## Waist Circumference

Waist circumference (cm) is measured to the nearest 0.1 cm using a non-elastic, flexible tape measure at the following time intervals: from baseline (T0) to 6-month follow-up (T3) (all participants), from baseline (T0) to 1 month (T1) (intervention group), from 1 month (T1) to 3 months (T2) (intervention group), and from 3 months (T2) to 6 months (T3) (intervention group). A reduction in waist circumference is associated with a more favourable cardiovascular outcomes compared to an increase in waist circumference, irrespective of weight loss status. The measurement site is defined as the midpoint between the lower margin

of the last palpable rib and the top of the iliac crest. Participants may keep their bras on but are asked to adjust their clothing to make measurements feasible. The research nurse palpates the area to ensure that measurements are taken at the correct location. The measurement is recorded while the participant is relaxed, exhaling, and standing with their torso and hips aligned, with weight evenly distributed over both feet. The measuring tape is held horizontally and is not pulled tight.

## Hip Circumference

Hip circumference (cm) is measured to the nearest 0.1 cm using a tape measure positioned at the largest part of the hip at the following time intervals: from baseline (T0) to 6-month follow-up (T3) (all participants), from baseline (T0) to 1 month (T1) (intervention group), from 1 month (T1) to 3 months (T2) (intervention group), and from 3 months (T2) to 6 months (T3) (intervention group). The measuring tape is held horizontally and should not be pulled tight. Participants are instructed to lower their trousers or skirts (but not their undergarments) and to stand with their arms hanging naturally along at their sides, adjacent to the torso and hips.

## Systolic and Diastolic Blood Pressure (SBP and DBP) Measurement

Blood pressure measurements are measured in mmHg, without decimal points, using portable blood pressure monitor (Welch-Allyn, USA). A reduction in blood pressure levels in the following time intervals: from baseline (T0) to 6 months (T3) (all participants), from baseline (T0) to 1 month (T1) (intervention group), from 1 month (T1) to 3 months (T2) (intervention group), and from 3 months (T2) to 6 months (T3) (intervention group), is considered a better outcome than vice versa. The research nurse ensures that the participant is adequately informed about the procedure and has emptied their bladder, if necessary. Following a 20-

minute period of rest in the supine position, blood pressure measurements are taken while the participant is seated on the examination table with the feet on the floor, with a supportive cushion under their arm. SBP and DBP pressure measurements are conducted in both arms, and the reading from the arm with the higher value is recorded. The cuff is placed directly against the skin, ensuring that no constrictive clothing is present above or below the cuff. The cuff fits snugly around the upper arm but not excessively tight; a finger is able to fit comfortably between the cuff and the skin. The palm of the participant's hand is facing upward. Participants with any confirmed SBP ≥ 200 mm Hg are referred to the emergency room, whilst those with a confirmed SBP ≥180 mm Hg are referred to primary health care services. In cases where the blood pressure readings are similar in both arms (i.e., a difference of less than 10 mmHg), the right arm is used for subsequent measurements. If there is a discrepancy in blood pressure readings between the arms, future measurements are consistently conducted in the arm with the higher blood pressure. It is essential to measure in the same arm for all follow-up assessments. Should the difference between the arms exceed 20 mmHg, further investigation is warranted. Blood pressure measurements are never taken in an arm with a fistula.

## Blood Samples

All blood specimens collected in this study are obtained from participants in a fasting state. The following blood samples are attained:

### *Liver status*

#### Plasma ASAT and Plasma ALAT

Liver status is assessed using plasma ASAT and plasma ALAT. The change of plasma ASAT and plasma ALAT is measured from baseline to the 6-month follow-up.

### *Metabolism*

#### Plasma glucose

Plasma glucose (mmol/L), which is a measure of glucose metabolism, is assessed. The change of plasma glucose is measured from baseline to the 6-month follow-up.

#### Plasma insulin

Also, plasma insulin (mmol/L) is assessed. The change of plasma insulin is measured from baseline to the 6-month follow-up.

#### Long term blood glucose control using blood glycated haemoglobin (HbA1C)

The change of HbA1C is measured from baseline (T0) to the 6-month follow-up (T3) (all participants). A normal HbA1C is below 42 mmol/mol (below 6%). Prediabetes is defined as 42-47 mmol (mol and 6.0 to 6.4%. Diabetes is defined as a HbA1C of 48 mmol/mol or two measurements exceeding 7%. Reduction of HbA1C values is identified by blood samples (for detailed information, see below).

### *Lipid status*

#### Plasma total-cholesterol

Reduction of total cholesterol levels is identified by blood samples. A total cholesterol below 200 mg/dL is defined as normal, from 200 to 239 mg/dL as borderline high, and 240 mg/dl and over is high. The change of total cholesterol is measured from baseline (T0) to the 6-month follow-up (T3) for the entire cohort of participants.

### Plasma low-density lipoprotein cholesterol (LDL-C) levels

Reduction of low-density lipoprotein cholesterol (LDL-C) levels (mmol/L) is identified by blood samples. LDL-C should be below 130 mg/dL. The change in LDL-C is measured from baseline (T0) to the 6-month follow-up (T3) across all participants.

### Plasma high-density lipoprotein cholesterol (HDL-C) levels

Rise of high-density lipoprotein cholesterol (HDL-C) levels (mmol/L) is identified by blood samples. A HDL-C value of exceeding 1.29 mmol/L is indicative of reduced stroke risk (1). HDL-C levels are measured from baseline (T0) to the 6-month follow-up (T3) to determine changes in the entire participant group.

### Plasma triglycerides

Triglyceride levels, expressed in mg/dL/, will be assessed via blood samples to identify any reductions. For adults, a healthy level is below 150 mg/dL. The change in triglycerides from baseline (T0) to the 6-month follow-up (T3) is measured across all participants.

## Blood Samples Procedures

If feasible, the research participant remains seated on the examination table. Participants are provided with information regarding the sample collection process. The fundamental principle is that the participants should fast from 22:00 hours (for tests conducted no earlier than 08:00 hours) on the evening preceding the blood test and may only consume a minimal quantity of water without additives. Furthermore, participants should refrain from using oral tobacco products or smoking, prior the test. All materials required for the sample collection are ensured to be readily accessible. The participant's identity is verified, preferably through the

presentation of an identification document, and by having the participant verbally confirm their name and personal identification number. Blood collection tubes are selected in accordance with the sampling guidelines established by the analysing laboratory. The additives within the tubes are specifically designed for various analyses and analytical methods. In our current study, when collecting multiple tubes simultaneously for Clinical Chemistry samples, we draw the heparin tube first, which may have a light green or green cap and may or may not contain gel. This is followed by the EDTA tube, identifiable by its purple cap, and then the glucose tube, which has a pink cap. For biobank samples, we draw a 10 mL Lithium Heparin tube, followed by a 10 mL EDTA tube, then a 10 mL serum tube, and finally a 5 mL EDTA tube. We ensure that each tube is filled to the indicated line. Additionally, we adhere to basic hygiene practices throughout the collection process. The site for venipuncture is carefully selected. To enhance venous dilation and increase blood flow, warmth is applied. Venepuncture is avoided in the ipsilateral arm of patients who have undergone due to concerns about the potential risk of lymphoedema and its effects on blood composition. Areas exhibiting oedema, hematoma, or extensive scarring are also avoided. Venipuncture is not performed on arteriovenous (AV) fistulas. Sterile gloves are put on prior to the procedure. The participant’s arm is positioned flat or slightly inclined downwards to prevent reflux. Local guidelines regarding skin disinfection are rigorously followed. Skin that is visibly soiled is washed and subsequently disinfected. For disinfection, a chlorhexidine solution (5 mg/mL) or an equivalent agent is employed. A minimum contact time of 30 seconds is required for the disinfectant to effectively air-dry. If necessary, a tourniquet is applied. The tourniquet is tightened for no longer than one minute. Should the tourniquet remain in place beyond this time, it is released and can be reapplied after a minimum of two minutes. Muscular activity, such as repeated fist clenching, is avoided during sample collection. Mechanical manipulation of the vein, including "tapping," is strictly avoided prior to sample

collection. Venipuncture is performed utilizing vacuum tubes according to The Handbook for Healthcare (2).

### *Plans for collection, laboratory evaluation and storage*

Biobank samples (10 mL Lithium Heparin, 10 mL EDTA, 10 mL serum, and 5 mL EDTA tubes) are collected from all participants at baseline and 6-month follow-up in addition to immediate clinical analyses. After processing, samples are aliquoted and stored at -80°C in the Sahlgrenska University Hospital Biobank with identification using only study ID numbers. These specimens are retained for future metabolomic and proteomic analyses in accordance with the Swedish Biobank Act and ethical approvals (reference numbers: 2023-00546-01 and 2023-07659-02). Participants will provide informed consent for storage and future use of biological specimens. Any analyses beyond those described in the current protocol will require additional ethical approval and application to the Swedish Ethical Review Authority.

## Physical Activity and Fitness Components

### *Saltin Grimby Physical Activity Scale (SGPALS)*

Self-reported physical activity level is assessed using the Saltin-Grimby Physical Activity Scale (SGPALS)(3-5), which is a four-level ordinal scale that ranges from one to four for assessing self-reported physical activity levels. The SGPALS consists of the following four categories: Sedentary (Level 1), Low Physical Activity (Level 2), Moderate Physical Activity (Level 3) and High Physical Activity (Level 4). The higher the value reported, the higher the individual's physical activity is rated on the scale. Originally, SGPALS refers to the past year, capturing habitual activity patterns, but the scale has evolved over time and can also refer to other time points (5). In this study, we refer to the last month. A higher self-reported physical

activity level at the 6-month follow-up (T3) compared with baseline (T0) is considered a better outcome than vice versa.

### *Aerobic fitness – Predicted maximal $VO_{2max}$*

Aerobic fitness, maximal oxygen uptake ($VO_{2max}$) capacity, is estimated by using the Ekblom-Bak test (6). An increase in predicted $VO_{2max}$ between baseline and 6-month follow-up (all participants) is considered a better outcome than a decrease in predicted $VO_{2max}$ over this period. Ekblom-Bak test is a submaximal cycling ergometer test developed to estimate maximal oxygen uptake ($VO_{2\ max}$) based on heart rate responses to incremental workloads. This test offers a practical, non-invasive method for assessing cardiorespiratory fitness in both clinical and research settings.

The research nurse ensures the cycling ergometer is properly calibrated according to the manufacturer's recommendations. The seat and handlebar positions for participant are adjusted to reach comfort and optimal pedalling efficiency. The participant is introduced to the Borg Rating of Perceived Exertion (RPE) (7, 8) scale prior to testing.

Participants must adhere to the following pre-test conditions: No heavy meals within three hours before the test, no smoking within two hours prior to the test, refrain from engaging in vigorous physical activity on the day of testing and the day prior and arrive in a calm and rested state, avoiding any stress or rushing to the testing location.

The Ekblom-Bak test protocol outlines tailored workloads for different fitness levels, with sex-specific considerations (6). The test begins with a 4-minute cycle at 30 watts, maintaining a steady pedalling rate of 60 revolutions per minute (rpm). Both pedalling speed and resistance are monitored to ensure consistency. Heart rates are recorded at four time points following the initiation of the test (T): 3 minutes and 15 seconds (T+3:15), 3 minutes

and 30 seconds (T+3:30), 3 minutes and 45 seconds (T+3:45) and 4 minutes (T+4:00) post-initiation (6).

This standard workload is followed by an individualized higher workload. The higher workloads for various activity levels, differentiated by sex, are delineated in the Ekblom-Bak test protocol, aiming to reach a heart rate range of 120–150 beat per minute (bpm for participants aged under 50 years or 110–140 bpm for participants aged 50 years and above (6). The participant cycles at this higher workload for 4 minutes, maintaining a pedalling rate of 60 rpm. The participant's heart rate is measured during the final minute as described for the standard workload phase. During the higher workload phase, the participant's RPE is assessed at two minutes. If the RPE is below or above the desired range, the resistance is adjusted accordingly: RPE < 10: Resistance is increased by 1 kilopond (kp); RPE 10–11: The resistance is increased by 0.5 kp; RPE 12–16: The current resistance is maintained; RPE ≥ 17: The test is stopped and rescheduled with a lower workload after 20 minutes rest. Record heart rate values, workload levels (in watts), and RPE scores are recorded during both phases of the test. The $VO_{2max}$ is calculated using the following equations:

For Men:

$$VO_{2max} = \mathrm{Exp}\left((2.04900 - 0.00858 \times \mathrm{Age}) - \left(0.90742 \times \frac{\Delta HR}{\Delta PO}\right) + (0.00178 \times \Delta PO) - (0.00290 \times HRstd)\right)$$

For Women:

$$VO_{2max} = \mathrm{Exp}\left((1.84390 - 0.00673 \times \mathrm{Age}) - \left(0.62578 \times \frac{\Delta HR}{\Delta PO}\right) + (0.00175 \times \Delta PO) - (0.00471 \times HRstd)\right)$$

where:

ΔHR = Change in heart rate between workloads.

ΔPO = Change in power output (watts) between workloads.

HRstd = Average heart rate during the standard workload phase.

This Ekblom-Bak protocol is validated for $VO_{2max}$ ranges of 19–62 mL·min$^{-1}$·kg$^{-1}$ for women and 24–76 mL·min$^{-1}$·kg$^{-1}$ for men. Applicable age range is 20–86 years for women and 20–84 years for men. Accuracy may be affected by deviations from pre-test conditions or using ergometers other than mechanically braked models.

### *Physical activity*

Time spent sedentary and in different physical activity intensity categories, overall Physical Activity Level (PAL) and compliance to the general recommendations on sedentary behaviour and physical activity set by the Public Health Agency of Sweden (9) are determined from accelerometer and fitness data. A decrease in sedentary time and increase in time at higher physical activity intensity categories and in PAL from baseline (T0) to 6-month follow-up (T3) (all participants) is considered a better outcome than an increase in sedentary time and a decrease in time at higher physical activity.

Participants are instructed to wear the accelerometer continuously for 24 hours per day over a period of seven days. The device is waterproof; however, it should be removed during showering, bathing, or swimming. The accelerometer should be positioned in a belt around the waist, situated on the right side above the hip bone, with the USB facing forward and the text facing inward. Participants are required to complete a diary entry each morning upon waking and each evening before going to bed. The accelerometer should be put on when the participant goes to bed in the evening before the measurement day one and be worn continuously for the full seven days. It should be removed upon waking on day eight. Upon completion of the measurement period, the accelerometer should be placed in a pre-paid envelope along with the diary.

Raw triaxial data from the Axivity AX3 accelerometer (Axivity Ltd, UK) is processed into a metrics of absolute physical activity intensity (milligravity, mg) with a

resolution of 3 seconds (epoch) using the 10 Hz Frequency Extended Method (10). Non-wear time is defined as 60 minutes of zero accelerometer output with allowance of up to 2 minutes of interruption below the sedentary threshold. A valid day was defined as at least 10 hours of wear time and a valid measurement as at least 4 valid days.

The mg metrics is translated into categories of absolute physical activity intensity and relative physical activity intensity (% $VO_{2max}$). The absolute physical activity intensity categories are defined from lab-based calibration studies (11) using Metabolic Equivalent of Task (MET) from measured O2 and CO2 as reference during rest and activities at various intensities, applying traditional cut-points of sedentary (<1.5 METs), light (1.5-2.9 METs), moderate (3.0-5.9 METs), vigorous (6.0-8.9 METs) and very vigorous physical activity intensity (≥9 METs) (12). In addition, field studies are used to determine a novel cut-point for moderate physical activity intensity based on the association pattern with cardiorespiratory fitness and cardiometabolic risk markers, arriving at approximately 4.5 METs [13]. The relative physical activity intensity categories are defined from these calibration studies expressing the reference measure in ml/kg/min O2 together with the estimated $VO_{2max}$ in the LI-PAD study, applying the cut-points of light (<46% $VO_{2max}$), moderate (46-63% $VO_{2max}$), vigorous (64-90% $VO_{2max}$) and very vigorous physical activity intensity (equal to or more than 91% $VO_{2max}$)(13). When comparing time spent in relative physical activity intensity categories between baseline and 6-month follow-up, the estimated $VO_{2max}$ at baseline is used for all calculations. If $VO_{2max}$ increases across the intervention and different $VO_{2max}$ values are used to determine time spent in the different physical activity intensity categories at baseline and at 6-month follow-up, it may seem that the study participant becomes less physically active (due to higher cut-points).

Based on the relative physical activity intensity, it may be feasible to assess whether participants meet the general recommendations for physical activity set by the Public

Health Agency of Sweden (9), which suggest 150-300 minutes/week of moderate intensity or 75-150 minutes/week of high intensity, or a combination of both (9). Specific recommendations from FYSS for individuals with overweight or obesity suggest ≥300 minutes/week of moderate intensity or 150 minutes/week of high intensity, or a combination thereof (14). It should be noted that national and international physical activity recommendations are not derived from accelerometer data but from self-reporting methods, which are not fully comparable (15). Therefore, conclusions on compliance should be drawn with cautiousness, until the day when the physical activity recommendations are developed based on accelerometer data. Relative physical activity intensity is used, as the commonly applied reference measure for moderate physical activity intensity 3 METs may be set too low and consequently overestimate compliance to physical activity recommendations (13). The 46% $VO_{2max}$ cut point would correspond to approximately 4.5 METs.

The calibrated MET-value for each epoch is used to estimate the PAL by calculating the mean MET of all epochs of accelerometer data collected over the 7 days measurement. Epochs of zero movement was assigned 1 MET intensity. The PAL is used together with REE to estimate the total energy expenditure (TEE) to determine energy need and energy intake (EI) to promote weight reduction. If accelerometer data is absent or insufficient, PAL values from the Nordic Nutrition Recommendations (NNR) is used (16):

- Bedbound or chairbound (not in a wheelchair) has a PAL of 1.1-1.2.
- Sedentary work with limited movement opportunities and no leisure activity corresponds to a PAL of 1.3-1.5.
- Sedentary work with some opportunities for movement and some leisure activity has a PAL of 1.6-1.7.
- Work that includes both standing and movement, or sedentary work with some movement and regular, almost daily leisure activity, is assigned a PAL of 1.8-

1.9. Very strenuous work or daily training at a competitive level is associated with a PAL of 2.0-2.4.

## Resting Energy Expenditure (REE)

REE is compared between baseline (T0) and 1-month follow-up (T1) (interventions), 1-month follow-up (T1) and 3-month follow-up (T2) (interventions), 3-month follow-up (T2) and 6-month follow-up (T3) (interventions), and finally between baseline (T0) and 6-month follow-up (T3) (all participants). An unchanged or increased REE is consider a better outcome than a decrease in REE. The concept is that a too restrictive energy intake with the aim of reducing body weight may lower REE and counteract metabolism of body fat and with reduction (17). Physical activity may increase muscle mass and consequently increase REE.

REE will be determined from direct measurement of gas exchange using COSMED Q-NRG device (COSMED Srl, Italy), more specific oxygen uptake ($O_2$) and the carbon dioxide ($CO_2$) during 20 minutes at rest in a lying position. The 15 last minutes of data will be included in the analysis. Of these minutes, the two to five minutes with the lowest data variation, with a variation of less 10%, will be used. Measured $O_2$ and $CO_2$ is translated into REE using the equation EE (kcal) = 3.94 x $O_2$ (l/min) + 1.1 x $CO_2$ (l/min) but also used to determine the respiratory quotient (RQ = $CO_2/O_2$) as an indicator of substrate utilization (fat versus carbohydrate). Prediction equations do not provide accurate and precise estimates of REE on overweight and obese individuals (18).

Prior to REE measurement, participants are instructed to adhere to a standardized protocol to minimize confounding factors: fasting state: no food consumption for four to five preceding the assessment; abstention form alcohol for a minimum of two hours prior to measurement; refraining from smoking, smokeless tobacco use, or nicotine consumption for at least two hours; avoidance of coffee or caffeine-containing beverages for a minimum of

four hours; abstention from moderate-intensity exercise for two hours and vigorous-intensity exercise for 14 hours prior to the assessment (19). Immediately before the measurement, participants are prompted to void their bladder if necessary. Additionally, adherence to the preparatory instructions is verbally confirmed with each participant to ensure protocol compliance and data integrity.

Participants should lie down on the bed immediately after the anthropometric measurements are completed to reach a resting state. A rest period of 10 to 20 minutes is required to achieve a stable measurement of resting energy expenditure (19).

A transparent canopy is placed over the participant's head, and a plastic cover is positioned around their body to prevent leakage. The canopy is adjusted to rest against the participant's chest and the slightly raised headboard of the bed. The airflow (Blower) is adjusted to ensure that the $FeCO_2$ remains between 0.7% and 1.5%. For larger participants, the airflow may need to be increased, while for smaller individuals, it may need to be decreased. Once $FeCO_2$ stabilizes between 0.7% and 1.5%, the research nurse/health promoter starts the recording to save data and conducts the measurement for 20 minutes. After completion of the test, the option to remove artefacts is selected to eliminate non-physiological data. The lowest five minutes are manually selected, and the data is saved. Variations should remain within ±10% for $O_2$ and $CO_2$. Given that the total time on the bed is 25–30 minutes, it is likely that the lowest five-minute segment will occur 10–15 minutes after start, as a 10 to 20 minutes resting period is necessary to achieve stable resting energy expenditure.

## Respiratory Quotient (RQ)

The respiratory quotient (RQ) is a metric that represents the ratio of carbon dioxide produced ($VCO_2$) to oxygen consumed ($VO_2$) during metabolic processes – it indicates the proportion

carbohydrate, fat and protein used as energy substrate. RQ is measured at baseline (all participants), at the 1-month (interventions) and the 3-month follow-up (interventions), and at the 6-month follow-up (all participants). A reduced or maintained lower RQ ($<0.8$) between the different time points is a positive outcome. RQ serves to assess the substrate utilization for energy production by the body both at rest and during physical activity (20). RQ values provide insights into energy metabolism as follows: 100% carbohydrates RQ $\approx$ 1.0, and 100% fat RQ $\approx$ 0.7.

## Energy Intake for Weight Reduction

The energy intake (EI) to promote a weight reduction of 5-10% at 6-month follow-up (21) is calculated as: EI (kcal/d) = REE x PAL – 500 (22). This EI is used for meal planning. The weight loss is monitored at 1-month (T1) and 3-month follow-up (T2). If the weight loss is slower than the expected 0.5kg/week, provided that the study participant is compliant to the prescribed EI, an additional 200 kcal/d energy deficit may be applied. Monitoring of changes in weight, REE and RQ is used to guide behavioural support and any adjustments of prescribed EI. A large reduction in REE between follow-ups, may indicate a too low EI (17).

## Upper and Lower Limb Strength and Functional Capacity

### *Hand grip strength*

The mean hand grip strength for both left and right hands is measured to one decimal place using a Jamar hand dynamometer (Patterson Medical, USA) (23), based on three attempts per hand. A better outcome in this study is defined as an improvement in handgrip strength, measured by increased kilograms, as evidenced by higher values at the six-month follow-up (T3) compared to baseline (T0) across all participants.

Before conducting the measurement, Jamar handgrip dynamometer is set to the second position from the inside of the handle. During the assessment, the participant is seated on a stool with an upright posture and with elbow in 90 flexion. The dynamometer is hold with a straight wrist, with the display facing forward (a maximum of 30 degrees of wrist dorsiflexion is acceptable, and the thumb should be positioned upward). During the test, the participant presses the handle as hard as he/she can until he/she is instructed to relax. The assessment comprises three attempts with each hand, alternating between the left and right hands. Whether the participants are right-handed or left-handed is registered in REDCap. If the participant is unable to prevent the display from tipping forward, the research nurse provides gentle support under the display. Once the participant is correctly positioned, the research nurse encourages the participant to press as hard as they can with left hand, harder, harder, and relax. The assessment continues identically with the right hand and is repeated two additional times resulting in a total of three attempts for each hand. All recorded values are documented, and the mean value is automatically calculated and registered in REDCap.

### *Sit-to-Stand Test (STS)*

Change in lower extremity functional mobility, strength and endurance is assessed using the Sit-to-stand test (24-27), the number of repetitions within 30 s is recorded, is performed from a chair with a seat height of 45 cm. A higher number of repetitions at the 6-month follow-up (T3) compared to the baseline (T0) is consider a better outcome.

### *Single-leg heel raises test*

Using the Single-leg heel raises test (28)  at a rate of 30 heel raises per minute on a 10° inclined wedge, primarily assesses muscular endurance and functional capacity of the plantar flexors. The measurements are conducted at baseline (T0) and at the 6-month follow-up (T3)

(all participants). The higher number of single-leg heel raises at the follow-up compared to baseline, the better the outcome compared to vice versa.

A metronome set at 60 beats per minute is used to ensure a consistent pace for all participants. The participant's head should reach the measuring rod on the wall with each heel raise. The wedge is attached to a 60 x 60 cm plywood board, with the wedge positioned at the centre of the board. The top edge of the wedge is 17 cm from the upper edge of the baseboard. The participant stands on their preferred leg (the same leg should be used in repeated tests). The initial heel raise height is determined by using a measuring rod by asking the participant to raise themselves as high as possible on the chosen leg. Record the height on the measuring rod in the protocol. The same height should be used for subsequent tests. The test is performed with appropriate footwear, unilaterally, with the test leg positioned straight on the wooden wedge, toes placed at the front edge of the wedge, and the foot centered on the wedge. The non-tested leg is kept flexed close to the standing leg. The participant may use their hands lightly against a wall or bar for balance, but without elevating himself/herself utilizing manual assistant. The test is terminated when the participant can no longer raise themselves to the initial height/flexes the knee/ pulls oneself up using one's hands or cannot maintain the set pace.

### *Unilateral dumbbell shoulder flexion test*

The seated dumbbell shoulder flexion test, performed with a straight arm between 0 and 90 degrees of shoulder flexion at a rate of 20 contractions per minute, primarily assesses muscular endurance and functional capacity of the shoulder flexor muscles (28). To keep all the participants at the same pace, a metronome is used. The more repetitions at the 6-month follow-up (T3) compared with baseline (T0) (all participants), the better the outcome.

The research nurse will record the selected side in REDCap, the same side must be used in repeated tests. The test involves moving the dumbbell with a straight arm up and down between 0° and 90° in the shoulder joint (the research nurse demonstrates the movement). A metronome set at 40 beats per minute, i.e., 20 contractions per minute, is used for pacing. The test will be stopped if the participant cannot reach 90° in the shoulder joint with a straight arm and/or perform the movement with a bent elbow or cannot maintain the set pace. The number of valid repetitions is recorded. The participant is permitted to practice the movement and pace several times using the arm not intended for assessment to familiarize themselves with the procedure. The research nurse will record the number of valid repetitions and log them into REDCap. If the participant is unable to manage the prescribed weight, the recorded measurement will be zero repetitions. For participants who have undergone sternotomy surgery, this test could be perform if at least 6 weeks postoperatively.

## Healthy Food Intake

Food intake is measured using the Meal-Q (29). Meal-Q is a web-based frequency questionnaire, which the participant completes using an iPad via a unique web-link provided by the research nurse. The questionnaire asks about dietary intake the past few months and is image-assisted for portion-size. The questions concern intake of 1) food items, dishes, and beverages, 2) energy and nutrients, including alcohol, 3) supplements, 4) meal patterns, and eating behaviour (e.g., restaurant visits, fast food, light products, use of cooking fat and salt). Nutrient intake is calculated using the Swedish Food Composition Database (30). Meal-Q is completed at baseline (T0) and the 6-month follow-up (T3) by all participants (i.e., by the interventions and the controls). In addition to investigate in meal, food and nutrient intake patterns, a Healthy Food Index (HFI) is applied as an overall metrics of a healthy food intake (31). It is calculated as the summed consumption frequences of 17 food items representing 5

food groups selected as indicators of healthy food choices: vegetables, fruits and berries, fatty fish, vegetable oils and nuts. The higher the HFI score, the better the outcome. Higher HFI score is associated with lower prevalence of coronary artery calcium in the SCAPIS pilot study (31).

## Lifestyle school 1: From risk-behaviour to health-behaviour

This lifestyle school is a two-hour group session to increase awareness of risk situations and risk factors interfering with weight reduction, and to replace them with health situations and health factors facilitating sustainable weight reduction. Theoretical knowledge is complemented by group discussions using a workbook with exercises, targeting topics like popular diets, stress, signal systems for hunger and satiety, sleep, motivation, goal setting, rewards, memory, habits, and solutions.

## Lifestyle school 2: From theory to the plate

This two-hour group session provides participants theoretical knowledge about the macro- and micro-nutrients, whilst guiding participants towards healthier dietary choices. Emphasis is placed on increasing intake of whole meals, vegetables and fruits, legumes, fish, and nuts, and less meat and processed foods containing excessive amounts of fats, salt, and sugar. This session also includes a practical exercise on the utilization of nutritional composition tables and portion size guidelines. This activity aims to assist participants in determining appropriate individual food and beverage quantities for their plates, with the objective of maintaining a healthy body mass.

## Lifestyle school 3: Find your Why

This two-hour group session is designed to increase awareness of externally motivated and automatic unhealthy actions in life. It guides the participants to reflect on these actions and transform them into new, intrinsic behaviours, with a focus on increasing physical activity and adopting to a healthier diet for weight reduction. Theoretical knowledge about diverse types of motivation (external vs internal) is combined with a workshop with practical exercises. The participants are exposed to several examples of externally motivated goals (e.g. "I must", "I should not") focusing on reducing or excluding unhealthy activities and foods (negative decisions) and have the task to change them into internally motivated goals (e.g. "I will", "I want") and including health activities and foods (positive decisions).

## Lifestyle school 4: Healthy food in your everyday life (cooking session)

The two-and-a-half-hour session in a teaching kitchen aims to support knowledge and skills in making healthy and practical meals and snacks for everyday use. The recipes used are adjusted to reduce energy content and intake of red meat, and increase intake of wholegrain, vegetables, fruits, pulses, and seeds in accordance with NNR2023 (38). Specific spices improve aroma and taste, making the meals more attractive, even for individuals used to meals with more meat and fat. The session starts with a theoretical background to the recipes and continues with cooking in teams of two participants. During the cooking, the health promoter discusses with the participants the content of the recipes and its purpose, and alterations in ingredients and spices. The session ends by eating the meals and to explore the experience of the participants.

## Lifestyle school 5: Physical activity and overweight/obesity (educational short film)

This educational short film, hosted within the mobile application, examines energy balance disruption and the national and global prevalence of overweight and obesity. It explores risk factors and health benefits of regular physical activity, discussing methodological approaches to aerobic exercise and strength training, including free weights, machines, and bodyweight exercises (32). The educational short film presents core exercise principles and concepts: intensity, duration, frequency, overload, progression, absolute and relative intensity, one-repetition maximum (1RM), dose-response relationships, training specificity, and physiological effects of regular physical activity. The educational short film explores participants' motivations and goal setting using the SMART (Specific, Measurable, Achievable, Relevant, Time-bound) criteria (33). The educational short film encourages introspection regarding exercise motivations, preferences, and potential barriers. It summarizes key points, emphasising physical activity's role in weight management and overall health. The educational short film presents evidence-based information aligned with scientific literature, (32) encouraging viewers to apply the content to their individual circumstances.

## Patient-Reported Outcome Measure (PROM)

As patient-reported outcome measures (PROM), the descriptive and the visual analogue part of the EuroQol Group's five Dimensions three Levels (EQ-5D-3L) consisting of two pages, the EQ-5D descriptive system and the EQ visual analogue scale (EQ VAS) are used (34, 35).

*The EQ descriptive system*

At baseline (T0) and the 6-month follow-up (T3), the research nurse requests all the participants (interventions and controls) to report their health status today using the EQ-5D-3L (34, 35) by marking the box corresponding to the most appropriate statement regarding severity level. There are three levels of severity: level 1: no problems (scored as 1), level 2: moderate problems (scored as 2), or level three: extreme problems (scored as 3) across five domains. The five domains relate to mobility, self-care, usual activities, pain/discomfort, and anxiety/depression. An increased proportion of participants reporting ‘no problem’ at the 6-month follow-up (T3) compared to baseline (T0) for each of the five dimensions is considered a more favourable outcome.

The scores for the five dimensions and three severity levels can be combined to create a five-digit number (a health state) representing one of 243 possible health states (health profiles) for the participant. For example, a health state of 11223 indicates no problems with mobility, no problems with self-care, some problems with usual activities, moderate pain/discomfort, and extreme anxiety/depression. A response of 11111 represents the lowest possible score, equivalent to no problems in any dimension an index value of 1 or perfect health. A health state can be converted to an index score by being matched to a corresponding value in a country-specific value set. Value sets are derived from population-based valuation studies, which use methods such as time trade-off or visual analogue scale (VAS) to obtain preferences for different health states. The resulting index score ranges from 1 (perfect health) to values below 0 (where 0 representing death) (36). A decrease in index score (down to 1) between baseline and the 6-month follow-up (all participants) is considered a better outcome than vice versa.

*The EQ visual analogue scale (EQ-VAS)*

Using an iPad, the participant is asked to rate their health today on EQ VAS, a vertical scale from 0 to 100 that best represents their assessment. The endpoints are labelled "Worst imaginable health" (=0) and "Best imaginable health" (=100). The EQ-VAS serves as a quantitative measure of health reflecting the participant's personal judgment. An increase in VAS score from baseline (T0) to the 6-month follow-up (T3) is considered a more favourable outcome compared to the inverse scenario.

## Exposures in the Built Environment

All participants (both the intervention group and controls) are invited to download the CLI lifestyle app. Participants are also asked to participate in Global Positioning System (GPS) tracking within the application and share GPS-data trough out the study period of 6 months. A change in spatial moment related to an increased PAL from baseline (T0) to the 6-month follow-up (T3) is considered a better outcome than vice versa. A change in exposure to unhealthy food outlets from baseline (T0) to the 6-month follow-up (T3) is considered a better outcome than vice versa.

The app collects data on date, time, height, location (GPS-coordinate – latitude and longitude with five m precision), and speed. The GPS-data is automatically transferred from participants phones via the mobile app to a secure server at the university several times a day. The app records all movement and sends a GPS-ping about every fifth second when the phone is in movement, and less often when the phone is not moving, when the app is in use or runs in the background. When the app is turned off, the app sends a GPS-ping about every 15 minutes.

Relevant information is extracted and clusters to identify points of interests from GPS trajectories at the individual and aggregate level are recognized. Data of interest is all

movements throughout the study period including daily path areas with buffer of all points or tracks, personalized maps of plotted tracks of movements including different travel modes, and composite measure of distances travelled and frequency at locations.

Given the lack of standard for optimal environmental measures (37), we will calculating multiple measures of exposures e.g. density of food outlets and facilities for physical activity, ratio of healthy/unhealthy food outlets, and temporal exposure along individuals GPS-routs. Exposures in the food environment include e.g., fast-food restaurants, full-service restaurants, bakeries, coffee shops, supermarkets, convenience stores, department stores, gas stations, fruit- and vegetable stores/markets. Exposures in the built environment for physical activity include e.g., green areas, in- and outdoor gyms, arenas for spontaneous sport, walking and bike lanes, and supplementary recreational spaces. The food and built environment are evaluated in relation to HFI score, PAL, cardiovascular risk factors, and in the degree of adherence to and adaption of behavioural modifications and interventional strategies.

## Process Evaluation

### *1. Number of implemented components*

The first process evaluation measure identifies the number of components implemented (intervention group). The various components that constitute the intervention is categorized as follows: Start meeting with medical feedback; physical activity and diet advises; and information and planning of intervention further intervention components; individual lifestyle counselling meeting of longer duration based on individual and objective measures of physical activity, aerobic fitness, energy requirement and support from a physical and activity and food app; supervised aerobic exercise; supervised strength training; entry pass for six months to Valhalla Swimming Hall in central Gothenburg; Lifestyle School with different

contents (group sessions) and an educational short film on physical activity and weight loss; information about other lifestyle tools and activities in the region, and follow-up measurement sessions of anthropometry, resting energy expenditure, respiratory quotient, blood pressure and blood samples.

### *2. Number of sessions of each intervention component delivered*

This second process evaluation quantifies the number of sessions delivered, to those in the intervention group, of eight intervention components (intervention group) according to: start meeting (1 session); individual lifestyle follow-up meeting (up to 8 shorter sessions of up to 30 minutes each); supervised aerobic exercise (twice a week); supervised strength training (twice a week); educational short film on physical activity and weight loss; Lifestyle School (five different); information about other lifestyle tools and activities at the start meeting; follow-up measurement of anthropometry, resting energy expenditure, respiratory quotient and blood pressure after 1 month, 3 months and 6 months.

### *3. Number of sessions of each intervention component received*

This third process evaluation quantifies the number of sessions of each of the following intervention components received (intervention group): Start meeting (1 session); individual lifestyle follow-up meeting (up to 8 shorter sessions of up to 30 minutes each); supervised aerobic exercise (twice a week); supervised strength training (twice a week); educational short film on physical activity and weight loss; Lifestyle School (five different); information about other lifestyle tools and activities at the start meeting; follow-up measurement of anthropometry, resting energy expenditure, respiratory quotient and blood pressure after 1 month, 3 months and 6 months.

### *4. Satisfaction with individual lifestyle counselling*

This fourth process evaluation attempts to provide an overall view of the participant satisfaction with the individual lifestyle counselling (intervention group). It combines categorical, open-ended (free text) questions and claims: “Have the counselling sessions helped you with lifestyle change for weight loss?” (Response options: Yes/No); If yes, ”How have the counselling sessions helped you with lifestyle change for weight loss?” (free text); “The counselling sessions have provided you with tools and strategies for lifestyle change for wight loss.” (Response options: Totally Agree, Somewhat Agree, Disagree); “Would you like more counselling sessions?” (Response options: Yes/No); and “What could have been improved with the counselling sessions?” (free text).

### *5. Satisfaction with Lifestyle School*

This process evaluation aims to provide an overall view of the participant satisfaction participating in the Lifestyle School. It combines open-ended (free text) and categorical questions, and statements: “Have you watched the Physical Activity and Weight Loss video found in the CLI lifestyle app?” (Response options: Yes/No). “Has the Lifestyle School helped you with lifestyle change for weight loss?” (Response options: Yes/No). If yes, ”How has the Lifestyle School helped you with lifestyle change for weight loss?” (free text); “Which of the Lifestyle School’s various events has been most beneficial in supporting your lifestyle changes for weight loss?” (Options: ‘From risk to healthy’, ‘From theory to plate’; ‘Find you why’; cooking; the educational short film ‘Physical Activity and weight loss’); “The Lifestyle School has provided you with lifestyle change tools and strategies for weight loss.” (Response options: Totally Agree, Somewhat Agree, Disagree); “Would you like more sessions of the Lifestyle School?” (Response options: Yes/No), and “What could have been improved with the Lifestyle School?” (free text).

## Evaluation of Aerobic Exercise, Strength Training in Group, Study Structure and Motivation for Continued Lifestyle Change

The evaluation of aerobic exercise comprises two prompts: 1. “This is what I thought was good about aerobic exercise”. (free text) 2. “This could have been done differently” (free text). Likewise, the evaluation of strength training also consists of two prompts: 1. “This is what I thought was good about strength training”. (free text). 2. “This could have been done differently”. (free text). Furthermore, evaluation of the study’s structure is evaluated with the following question: “Do you have any suggestions for improving the study's structure?” (free text. Finally, evaluation of the participants motivation for continued lifestyle change is conducted by prompting the participant to rate their motivation on a scale from 1 (not motivated at all) to 10 (very motivated).

## References


1. Sacco RL, Benson RT, Kargman DE, Boden-Albala B, Tuck C, Lin IF, et al. High-density lipoprotein cholesterol and ischemic stroke in the elderly: the Northern Manhattan Stroke Study. Jama. 2001;285(21):2729-35.
2. Tillmar L. Vårdhandboken - Blodprov (Ventipuncture) https://www.vardhandboken.se/undersokning-och-provtagning/blodprov/2021, rev. 2024 [
3. Saltin B, Grimby G. Physiological analysis of middle-aged and old former athletes. Comparison with still active athletes of the same ages. Circulation. 1968;38(6):1104-15.
4. Rödjer L, Jonsdottir IH, Rosengren A, Björck L, Grimby G, Thelle DS, et al. Self-reported leisure time physical activity: a useful assessment tool in everyday health care. BMC Public Health. 2012;12:693.

5. Grimby G, Börjesson M, Jonsdottir IH, Schnohr P, Thelle DS, Saltin B. The "Saltin-Grimby Physical Activity Level Scale" and its application to health research. Scand J Med Sci Sports. 2015;25 Suppl 4:119-25.

6. Ekblom-Bak E, Björkman F, Hellenius ML, Ekblom B. A new submaximal cycle ergometer test for prediction of VO2max. Scand J Med Sci Sports. 2014;24(2):319-26.

7. Borg G. Perceived exertion as an indicator of somatic stress. Scand J Rehabil Med. 1970;2(2):92-8.

8. Borg GA. Psychophysical bases of perceived exertion. Med Sci Sports Exerc. 1982;14(5):377-81.

9. Folkhälsomyndigheten. Riktlinjer och rekommendationer för fysisk aktivitet och stillasittande. Folkhälsomyndigheten; 2023.

10. Fridolfsson J, Arvidsson D, Ekblom-Bak E, Ekblom Ö, Bergström G, Börjesson M. Accelerometer-measured absolute versus relative physical activity intensity: cross-sectional associations with cardiometabolic health in midlife. BMC Public Health. 2023;23(1):2322.

11. Fridolfsson J, Börjesson M, Buck C, Ekblom Ö, Ekblom-Bak E, Hunsberger M, et al. Effects of Frequency Filtering on Intensity and Noise in Accelerometer-Based Physical Activity Measurements. Sensors (Basel). 2019;19(9).

12. Migueles JH, Cadenas-Sanchez C, Ekelund U, Delisle Nyström C, Mora-Gonzalez J, Löf M, et al. Accelerometer Data Collection and Processing Criteria to Assess Physical Activity and Other Outcomes: A Systematic Review and Practical Considerations. Sports Med. 2017;47(9):1821-45.

13. Fridolfsson J, Ekblom-Bak E, Ekblom Ö, Bergström G, Arvidsson D, Börjesson M. Fitness-related physical activity intensity explains most of the association between

accelerometer data and cardiometabolic health in persons 50-64 years old. Br J Sports Med. 2024.

14. Dohr I-M, Jansson, E., Börjesson, M., Hagströmer, M. Yrkesföreningar för fysisk aktivitet. FYSS 2021. fysisk aktivitet i sjukdomsprevention och sjukdomsbehandling. 4e upplagan ed: Läkartidningen förlag; 2021. 500 p.

15. Troiano RP, McClain JJ, Brychta RJ, Chen KY. Evolution of accelerometer methods for physical activity research. Br J Sports Med. 2014;48(13):1019-23.

16. Blomhoff R, Andersen, R., Arnesen, E. K., Christensen, J. J., Eneroth, H., Erkkola, M., Gudanaviciene, I., Halldórsson, Þ. I., Høyer-Lund, A., Lemming, E. W., Meltzer, H. M., Pitsi, T., Schwab, U., Siksna, I., Þórsdóttir, I., & Trolle, E. . Nordic Nutrition Recommendations 2023: Integrating Envirnmental Aspects.; 2023.

17. Redman LM, Heilbronn LK, Martin CK, de Jonge L, Williamson DA, Delany JP, et al. Metabolic and behavioral compensations in response to caloric restriction: implications for the maintenance of weight loss. PLoS One. 2009;4(2):e4377.

18. Madden AM, Mulrooney HM, Shah S. Estimation of energy expenditure using prediction equations in overweight and obese adults: a systematic review. J Hum Nutr Diet. 2016;29(4):458-76.

19. Compher C, Frankenfield D, Keim N, Roth-Yousey L. Best practice methods to apply to measurement of resting metabolic rate in adults: a systematic review. J Am Diet Assoc. 2006;106(6):881-903.

20. Miles-Chan JL, Dulloo AG, Schutz Y. Fasting substrate oxidation at rest assessed by indirect calorimetry: is prior dietary macronutrient level and composition a confounder? Int J Obes (Lond). 2015;39(7):1114-7.

21. Jensen MD, Ryan DH, Apovian CM, Ard JD, Comuzzie AG, Donato KA, et al. 2013 AHA/ACC/TOS guideline for the management of overweight and obesity in adults: a

report of the American College of Cardiology/American Heart Association Task Force on Practice Guidelines and The Obesity Society. Circulation. 2014;129(25 Suppl 2):S102-38.

22. Cooney C, Daly E, McDonagh M, Ryan L. Evaluation of Measured Resting Metabolic Rate for Dietary Prescription in Ageing Adults with Overweight and Adiposity-Based Chronic Disease. Nutrients. 2021;13(4).

23. Hamilton GF, McDonald C, Chenier TC. Measurement of grip strength: validity and reliability of the sphygmomanometer and jamar grip dynamometer. J Orthop Sports Phys Ther. 1992;16(5):215-9.

24. McKay MJ, Baldwin JN, Ferreira P, Simic M, Vanicek N, Burns J. Reference values for developing responsive functional outcome measures across the lifespan. Neurology. 2017;88(16):1512-9.

25. Arimoto A, Ishikawa S, Tadaka E. Empirical study of the 30-s chair-stand test as an indicator for musculoskeletal disorder risk of sedentary behaviour in Japanese office workers: a cross-sectional empirical study. BMJ Nutr Prev Health. 2021;4(1):158-65.

26. Jones CJ, Rikli RE, Beam WC. A 30-s chair-stand test as a measure of lower body strength in community-residing older adults. Res Q Exerc Sport. 1999;70(2):113-9.

27. Lázaro-Martínez S, Orueta-Jiménez TJ, Arias-Vázquez PI, Castillo-Avila RG, Tovilla-Zárate CA, Hernández-Gil KDC, et al. Reproducibility and safety of the 30" sit to stand test in individuals with obesity and cardiovascular risk factors. Obes Res Clin Pract. 2022;16(6):533-5.

28. Cider A, Carlsson S, Arvidsson C, Andersson B, Sunnerhagen KS. Reliability of clinical muscular endurance tests in patients with chronic heart failure. Eur J Cardiovasc Nurs. 2006;5(2):122-6.

29. Christensen SE, Möller E, Bonn SE, Ploner A, Wright A, Sjölander A, et al. Two new meal- and web-based interactive food frequency questionnaires: validation of energy and macronutrient intake. J Med Internet Res. 2013;15(6):e109.

30. Livsmedelsverket. The Swedish Food Agency food database: Livsmedelsverket; 2024 [

31. Gripeteg L, Arvidsson D, Johannesson E, Larsson C, Sjöberg A, Angerås O, et al. Concomitant Associations of Healthy Food Intake and Cardiorespiratory Fitness With Coronary Artery Calcium. Am J Cardiol. 2018;122(4):560-4.

32. Mattson M, Jansson, E., Hagströmer, M. Fysisk aktivitet - begrepp och principer. In: (YFA) YfFA, editor. FYSS 2021: Fysisk aktivitet i sjukdomsprevention och sjukdomsbehandling. Stockholm: Yrkesföreningar för Fysisk Aktivitet (YFA); 2021. p. 23-36.

33. Bovend'Eerdt TJ, Botell RE, Wade DT. Writing SMART rehabilitation goals and achieving goal attainment scaling: a practical guide. Clin Rehabil. 2009;23(4):352-61.

34. EuroQol--a new facility for the measurement of health-related quality of life. Health Policy. 1990;16(3):199-208.

35. Brooks R. EuroQol: the current state of play. Health Policy. 1996;37(1):53-72.

36. Hermann Z, Péntek M, Gulácsi L, Kopcsóné Németh IA, Zrubka Z. Measuring the acceptability of EQ-5D-3L health states for different ages: a new adaptive survey methodology. Eur J Health Econ. 2022;23(7):1243-55.

37. Siddiqui NZ, Wei L, Mackenbach JD, Pinho MGM, Helbich M, Schoonmade LJ, et al. Global positioning system-based food environment exposures, diet-related, and cardiometabolic health outcomes: a systematic review and research agenda. Int J Health Geogr. 2024;23(1):3.

38. Nordic Council of Ministers. Nordic Nutrition Recommendations 2023: Integrating Environmental Aspects. Nordic Council of Ministers; 2023
