# Supplementary material for: An individualised Lifestyle Intervention with Physical Activity and Diet in individuals with overweight and obesity (LI-PAD)—study protocol of a 6-month randomised controlled study {1a}
Source: Trials. 2026 Mar 16;27:232. doi: 10.1186/s13063-026-09606-6 (PMC13019878; doi:10.1186/s13063-026-09606-6)
Supplement: Supplementary file 2 — Additional file 2. [file 13063_2026_9606_MOESM2_ESM.docx]

**Table 1.** Schedule and the key components of the enrolment, interventions, and

Assessments

|  | **Study period** | | | | | | |
| --- | --- | --- | --- | --- | --- | --- | --- |
| **Timepoint** |  | **T0** |  |  | **T1** | **T2** | **T3** |
|  | **Recruit-ment and scree-ning** | **Baseline assess-ments** | **Rando-mization** | **Initial meet-ing** | **Follow-ups at one, three- and six-months post-study start.** | | |
| **ENROLMENT** |  |  |  |  |  |  |  |
| **Eligibility screen** |  |  |  |  |  |  |  |
| Screening 1  Request for study participation  Per post and QR-code | ✓ |  |  |  |  |  |  |
| Screening 2  By phone and baseline | ✓ | ✓ |  |  |  |  |  |
| **Informed consent** |  | ✓ |  |  |  |  |  |
| **Allocation** |  |  | ✓ |  |  |  |  |
| **INTERVENTIONS** |  |  |  |  |  |  |  |
| A 6-month, individualized intervention designed to optimize behaviour change, incorporating objective assessments and a comprehensive portfolio of activities, described in manuscript |  |  |  | ✓ | ✓ | ✓ | ✓ |
| **ASSESSMENTS** |  |  |  |  |  |  |  |
| **Anthropometry I** |  |  |  |  |  |  |  |
| Date of assessment |  | ✓ |  | ✓ | ✓ | ✓ | ✓ |
| Start time of assessment |  | ✓ |  | ✓ | ✓ | ✓ | ✓ |
| ID check | ✓ | ✓ |  | ✓ | ✓ | ✓ | ✓ |
| Signed informed consent |  | ✓ |  |  |  |  |  |
| Age (years) | ✓ | ✓ |  |  |  |  |  |
| Sex  Woman/Man, n (%) | ✓ | ✓ |  |  |  |  |  |
| Check of preparations  Yes/No, n (%) |  | ✓ |  |  | ✓ | ✓ | ✓ |
| Weight (kg), ^a^ self-reported | ✓^a^ | ✓ |  |  | ✓ | ✓ | ✓ |
| Length (cm), ^a^ self-reported  self-reported | ✓^a^ | ✓ |  |  |  |  | ✓ |
| BMI (kg/m^2^), ^a^ self-reported | ✓^a^ | ✓ |  |  | ✓ | ✓ | ✓ |
| Waist circumference (cm) |  | ✓ |  |  | ✓ | ✓ | ✓ |
| Hip circumference (cm) |  | ✓ |  |  | ✓ | ✓ | ✓ |
| Waist-to-hip ratio |  | ✓ |  |  | ✓ | ✓ | ✓ |
| **Tests part 1** |  |  |  |  |  |  |  |
| No meal at the latest 4-5 hrs.  Yes/No, n (%) |  | ✓ |  |  | ✓ | ✓ | ✓ |
| No alcohol for the last 2 hrs.  Yes/No, n (%) |  | ✓ |  |  | - ✓ | ✓ | ✓ |
| No smoking, snuffing,  Nicotine the latest 2 hrs.  Yes/No, n (%) |  | ✓ |  |  | ✓ | ✓ | ✓ |
| No coffee or caffein the  latest 4 hours  Yes/No, n (%) |  | ✓ |  |  | ✓ | ✓ | ✓ |
| No moderate or vigorous  exercise for the latest 2 hrs.  Yes/No, n (%) |  | ✓ |  |  | ✓ | ✓ | ✓ |
| **Clinical tests 1** |  |  |  |  |  |  |  |
| Resting energy expenditure (kcal/24 hrs.) |  | ✓ |  |  | ✓ | ✓ | ✓ |
| Respiratory Quotient |  | ✓ |  |  | ✓ | ✓ | ✓ |
| **Anthropometry part 2** |  |  |  |  |  |  |  |
| Regular pulse  Yes/No, n (%) |  | ✓ |  |  |  |  | ✓ |
| Systolic blood pressure (mm Hg) |  | ✓ |  |  | ✓ | ✓ | ✓ |
| Diastolic blood pressure (mm Hg) |  | ✓ |  |  | ✓ | ✓ | ✓ |
| **Tests part 2** |  |  |  |  |  |  |  |
| Fitness test - Ekblom-Bak cycle ergometer test using cycle ergometer Monark Ergomedic 828 E |  | ✓ |  |  |  |  | ✓ |
| Handgrip strength using Jamar hand dynamometer |  | ✓ |  |  |  |  | ✓ |
| Mean handgrip strength right hand, based on three attempts (kg) |  | ✓ |  |  |  |  | ✓ |
| Mean handgrip strength right hand, based on three attempts (kg) |  | ✓ |  |  |  |  | ✓ |
| Mean handgrip strength left hand, based on three attempts (kg) |  | ✓ |  |  |  |  | ✓ |
| **Lower limb functional strength and endurance** |  |  |  |  |  |  |  |
| Sit-to-stand test  (Number of repetitions in 30 s) |  | ✓ |  |  |  |  | ✓ |
| **Upper limb functional capacity and muscular endurance** |  |  |  |  |  |  |  |
| Shoulder flexion 0-90 degrees using a dumbbell, of 2 kg for women and of 3 kg for men (Number of repetitions with 40 beats/min) |  | ✓ |  |  |  |  | ✓ |
| **Plantar flexor strength** |  |  |  |  |  |  |  |
| Heel rises (Number of repetitions at a pace of 30 heel rises/min) |  | ✓ |  |  |  |  | ✓ |
| **Accelerometer**  **introduction given** |  | ✓ |  |  |  |  | ✓ |
| **Questionnaires** |  |  |  |  |  |  |  |
| **Self-rated physical activity level using the Saltin Grimby Physical Activity Scale (SGPALS)**, (n %). Scale: 1-4; |  | ✓ |  |  |  |  | ✓ |
| 1=Physically inactive, 2=Some light physical  activity  3=Regular physical activity and training  4=Regular hard physical training for competitive sports |  |  |  |  |  |  |  |
| **Meal-Q web-based food frequency questionnaire**, past few months, image-assisted for portion-size |  | ✓ |  |  |  |  | ✓ |
| Components:  1. Food items, dishes, beverages  2. Energy and nutrients, including alcohol  3. Supplements  4. Meal patterns  5. Eating behaviour, e.g. restaurant, fast food, light products |  |  |  |  |  |  |  |
| **Health-related quality of life using the EuroQol** five dimensions (Mobility, Self-Care, Usual Activities, Pain/Discomfort, Anxiety/Depression) and three levels: |  | ✓ |  |  |  |  | ✓ |
| 1. No problem 2. Moderate problems 3. Severe problems |  |  |  |  |  |  |  |
| EQ-5D-3L index score  (0-1) |  | ✓ |  |  |  |  | ✓ |
| EQ-VAS (Scale: 0-100, 0=worst health you can imagine, 100=the best health you can imagine) |  | ✓ |  |  |  |  | ✓ |
| **Motivation** |  |  |  |  |  |  |  |
| How important is it for you to perform this lifestyle change? (Scale: 0-10, 0=not at all, 10= extremely important) |  | ✓ |  |  |  |  | ✓ |
| How important is it to you to stick to the lifestyle change you started/implemented here in the research project? (Scale: 1-5, 1=not correct at all, 5=completely correct |  | ✓ |  |  |  |  | ✓ |
| It is important for me to make the lifestyle change (Scale: 1-5, 1=not correct at all, 5=completely correct |  | ✓ |  |  |  |  | ✓ |
| I’m unsure why I should make this lifestyle change (Scale: 1-5, 1=not correct at all, 5=completely correct |  | ✓ |  |  |  |  | ✓ |
| I feel awful if I do not make the lifestyle change (Scale: 1-5, 1=not correct at all, 5=completely correct |  | ✓ |  |  |  |  | ✓ |
| I make the lifestyle change because I want to (Scale 1-5, 1=not correct at all, 5=completely correct |  | ✓ |  |  |  |  | ✓ |
| How confident are you that you can make/implement this lifestyle change? Scale: 0-10, =extremely confident  n (%) |  | ✓ |  |  |  |  | ✓ |
| **Aches and pains** |  |  |  |  |  |  |  |
| Do you have any ongoing rest pain that affects your ability to be physically active?  Yes/No, n (%) |  | ✓ |  |  |  |  | ✓ |
| Rest pain >3 months  Yes/No, n (%) |  | ✓ |  |  |  |  | ✓ |
| If rest pain, how much  pain do you have? Scale 0-10,  0=none, 10=worst imaginable  Score |  | ✓ |  |  |  |  | ✓ |
| Do you have any ongoing load-related pain that affects your ability to be physically active?  Yes/No, n (%)) |  | ✓ |  |  |  |  | ✓ |
| If yes, load-related pain more than 3 months?  Yes/No, n (%) |  | ✓ |  |  |  |  | ✓ |
| If load related pain, how painful is it? Scale 0-  10, 0=none, 10=worst imaginable (score) |  | ✓ |  |  |  |  | ✓ |
| **Other questions** |  |  |  |  |  |  |  |
| **Support** |  |  |  |  |  |  |  |
| Do you have someone in your environment who supports you to be physically active?  Yes/No, n (%) |  | ✓ |  |  |  |  | ✓ |
| **Medications** |  |  |  |  |  |  |  |
| Do you take any medications regularly (every day)? Applies to both prescription and over-the-counter medications Yes/No, n (%)  If yes, specify |  | ✓ |  |  |  |  | ✓ |
| Number of medicines per day (n) |  | ✓ |  |  |  |  | ✓ |
| **Diabetes mellitus** |  |  |  |  |  |  |  |
| Prescence of diabetes Yes/No, n (%) |  | ✓ |  |  |  |  | ✓ |
| **Other non-cardiometabolic diagnoses** |  |  |  |  |  |  |  |
| Other diagnoses (non-cardiometabolic)  Yes/No, n (%) |  | ✓ |  |  |  |  | ✓ |
| **Smoking** |  |  |  |  |  |  |  |
| Never  Quit smoking >1 month  ago  Smoking or stopped smoking < 1 month ago  n (%) |  | ✓ |  |  |  |  | ✓ |
| **Highest educational level** |  |  |  |  |  |  |  |
| University  High school  Elementary school  Elementary school  Non-completed  Unknown/not stated  n (%) |  | ✓ |  |  |  |  | ✓ |
| **Blood samples** |  |  |  |  |  |  |  |
| *Infection status* |  |  |  |  |  |  |  |
| Plasma CRP  Mg/L |  | ✓ |  |  |  |  | ✓ |
| *Liver function status* |  |  |  |  |  |  |  |
| Plasma ASAT  µkat/L |  | ✓ |  |  |  |  | ✓ |
| Plasma ALAT  µkat/L |  | ✓ |  |  |  |  | ✓ |
| *Metabolism* |  |  |  |  |  |  |  |
| Plasma glucose  mmol/L |  | ✓ |  |  |  |  | ✓ |
| HbA1c  mmol/L |  | ✓ |  |  |  |  | ✓ |
| Plasma insulin  mmol/L |  | ✓ |  |  |  |  | ✓ |
| *Lipid profile* |  |  |  |  |  |  |  |
| Plasma total cholesterol  mmol/L |  | ✓ |  |  |  |  | ✓ |
| LDL-cholesterol  mmol/L |  | ✓ |  |  |  |  | ✓ |
| HDL-cholesterol  mmol/L |  | ✓ |  |  |  |  | ✓ |
| Triglycerides  mmmol/L |  | ✓ |  |  |  |  | ✓ |
| Biobank (four tubes)  Metabolomics  Proteomics |  | ✓ |  |  |  |  | ✓ |
| **Accelerometer data**  Using Axivity AX3 accelerometer (Axivity Ltd, UK) |  |  |  |  |  |  |  |
| Valid days  N (%) |  | ✓ |  |  |  |  | ✓ |
| Time sedentary  (i.e., <1.5 METS)  Mean (SD) min/day |  | ✓ |  |  |  |  | ✓ |
| Time moderate physical activity (46-63% VO_2_ max)  Mean (SD) min/day |  | ✓ |  |  |  |  | ✓ |
| Time moderate and vigorous physical activity (≥4 METs)  Mean (SD) min/day |  | ✓ |  |  |  |  | ✓ |
| Physical activity level (i.e., total energy expenditure/basal metabolic rate) |  | ✓ |  |  |  |  | ✓ |
| **Energy** |  |  |  |  |  |  |  |
| Resting energy expenditure  Kcal/24 hrs |  | ✓ |  |  |  |  | ✓ |
| Value from accelerometer format |  |  |  |  |  |  |  |
| Total energy expenditure, kcal/24 hrs. |  | ✓ |  |  |  |  | ✓ |
| Energy intake for weight reduction, kcal/24 hrs. |  | ✓ |  |  |  |  | ✓ |
| **Risk assessment** |  |  |  |  |  |  |  |
| Excluded before the risk assessment  Yes/No, n (%) |  | ✓ |  |  |  |  | ? |
| Known aorta stenosis  Yes/No, n (%) |  | ✓ |  |  |  |  |  |
| Symptomatic angina  Yes/No, n (%) |  | ✓ |  |  |  |  |  |
| Ongoing infection  Yes/No, n (%) |  | ✓ |  |  |  |  |  |
| **Relative contra indications** |  |  |  |  |  |  |  |
| Irregular pulse  Yes/No, n (%) |  | ✓ |  |  |  |  |  |
| High blood pressure (≥200 mmHg)  Yes/No, n (%) |  | ✓ |  |  |  |  |  |
| Blood pressure ≥180 mm Hg)  Yes/No, n (%) |  | ✓ |  |  |  |  |  |
| fB glucose high (≥6.0 mmol/L)  Yes/No, (%) |  | ✓ |  |  |  |  |  |
| Known diabetes  Yes/No, n (%) |  | ✓ |  |  |  |  |  |
| Newly detected diabetes (fB Glucose ≥6.5 mmol/L)  Yes/No, n (%) |  | ✓ |  |  |  |  |  |
| High glucose, DM  Yes/No, n (%) |  | ✓ |  |  |  |  |  |
| f Cholesterol high (≥6.9 mmol/L)  Yes/No, n (%) |  | ✓ |  |  |  |  |  |
| Familial hypercholesterolemia  Yes/No, n (%) |  | ✓ |  |  |  |  |  |
| Known high cholesterol  Yes/No, (%) |  | ✓ |  |  |  |  |  |
| Newly detected high cholesterol  Yes/No, n (%) |  | ✓ |  |  |  |  |  |
| Other tests that need action  Yes/No, n (%) |  | ✓ |  |  |  |  |  |
| SCORE 2  Low risk (<5%)  Medium risk (5-<20%  High risk (≥20%)  N (%) |  | ✓ |  |  |  |  |  |
| SCORE 2 + Diabetes Mellitus  Yes/No, (%) |  | ✓ |  |  |  |  |  |
| Ongoing symptoms  Yes/No, n (%)  If yes, specify |  | ✓ |  |  |  |  |  |
| Other medical history  Yes/No, n (%)  If yes, specify |  | ✓ |  |  |  |  |  |
| Relative contraindication for participation  Yes/No, n (%) |  | ✓ |  |  |  |  |  |
| Referral to another medical outpatient unit  Yes/No, n (%) |  | ✓ |  |  |  |  |  |
| Approval for participation  Yes/No, (n%) |  | ✓ |  |  |  |  |  |
| **Start - Initial meeting**  **1a). Plan for achieving 300 minutes of aerobic physical activity (PA) of moderate intensity/w** |  |  |  |  |  |  |  |
| Aerobic PA (self) (min)  Aerobic PA (in group) (min)  PA in water (including) (min) |  |  |  | ✓ |  |  |  |
| **1b) Plan for muscle strength training** |  |  |  | ✓ |  |  |  |
| Muscle strength training (self)  Muscle strength training (in group) |  |  |  | ✓ |  |  |  |
| **2) Plan for diet/calorie restriction and behavioural change** |  |  |  | ✓ |  |  |  |
| **Diet plan activities**  Diet (self)  Diet energy reduced  Diet diary  Diet scheme  **Intervention activities**  Health counselling sessions and follow-up conversations  Telephone follow-up  Nudging  CLI Lifestyle Intervention App  Paper-based dietary recording  Online health coaching (Hälsocoach online)  Lifestyle tool (Livsstilsverktyget)  Lifestyle school (Livsstilsskola) |  |  |  | ✓ |  |  |  |
